# Making ties and social identities: Drawing connections between PPNB communities as based on shell bead typology

**DOI:** 10.1371/journal.pone.0289091

**Published:** 2023-11-28

**Authors:** Heeli C. Schechter, David S. Reese, Daniella E. Bar-Yosef Mayer, A. Nigel Goring-Morris

**Affiliations:** 1 Institute of Archaeology, The Hebrew University of Jerusalem, Jerusalem, Israel; 2 Peabody Museum of Natural History, Yale University, New Haven, Connecticut, United States of America; 3 The Steinhardt Museum of Natural History and the Institute of Archaeology, Tel Aviv University, Tel Aviv, Israel; 4 Peabody Museum of Archaeology and Ethnology, Harvard University, Cambridge, Massachusetts, United States of America; University of California Santa Cruz, UNITED STATES

## Abstract

People tend to belong to multiple social circles, which construct and reflect a person’s social identity. Group affiliation is embodied and may be expressed by personal adornment. Personal adornment in general has multiple functions in human societies, among them the assimilation and transmission of different aspects of personal and collective, social and cultural identity. Beads in general, including shell beads, often constitute parcels of composite adornment, and as such are used in different configurations to portray these messages. The shared use of similar bead types by different individuals and communities indicates the mutual affiliation of the sharing parties to the same cultural circles and reflects social ties and relationships. The Pre-Pottery Neolithic B (PPNB) period in the Levant is a time of pivotal changes to human lifeways necessitating profound adjustments in all aspects of life, including social relations and networks. Here we use the shell bead assemblage from the cultic-mortuary aggregation site of Kfar HaHoresh, in comparison to shell bead assemblages from multiple other sites in the Levant, as a proxy for the exploration of local and regional networks and connections between PPNB communities. Multivariate analyses of shell bead type distribution patterns across the Levant demonstrate that some types were widely shared among different communities, characterising different geographic regions, while others were rare or unique, highlighting relationships between sites and regions, which are occasionally independent of geographic proximity. Specific occurrences of shared shell bead types between Kfar HaHoresh and compared sites further illuminate the web of connections between PPNB communities in the Levant and the varying breadths of sharing-patterns reflect the hierarchical nature of the underlying social circles. Outlining these widening social affiliations sheds light on the complex structure of Neolithic social identity.

## Introduction

Humans strive for a sense of social belonging as an innate feature of human nature. We identify ourselves with certain social groups (ethnic, political, genealogical, gendered, occupational, age-based, etc.) and disassociate ourselves from others, assuming the different roles these associations entail. Different theories in social psychology, such as Social Identity and Social Categorization [1–4] examine and explain these tendencies, and the social processes and phenomena deriving from them, and provide a broad theoretical basis for interpreting and understanding different human social behaviours [5–9]. These theories suggest that concepts of self-identity are constructed according to personal, relational, and collective levels of self-definition, which create a hierarchy of identities. Personal and social aspects of self-definition and identity may be differentiated, or positioned on a spectrum, allowing people to be defined and understood not only as individuals but also as belonging to certain wider social categories. Individuals categorize themselves as belonging to multiple social groups [i.e. 10] and group memberships are internalized as relevant aspects of the self-concept. Group memberships are also externalized and accentuated, in order to strengthen in-group cohesiveness and out-group differentiation [as in 11, 12], by the use of different means, for example, personal adornment or ornamentation of various artifacts such as basketry.

Personal adornment, in our case, involves the wearing on the body of different artefacts, imbued with social and cultural meaning. The use of personal adornment constitutes an embodiment of identity; an introverted bodily construction of identity, on the one hand, and its extroverted communication, on the other [13–17]. On a social scale it is designed to negotiate and transmit messages concerning different aspects of peoples’ personal and social identity [e.g. 12, 18–22]. The shared use of specific items of personal adornment by different members of a social group, expresses their mutual affiliation to one another and to the social group at large [23–25].

From an archaeological perspective, items of adornment found in archaeological sites, used to ornament humans, as well as animals or associated equipment (i.e. beads sewn on presumed headgear and other fabrics from Nahal Hemar Cave [26]), reflect the choices and actions of individuals operating within the social and cultural contexts of the community using the site. Recording the specific types of adornment found and tracking the different trends of where and when each type appears, may illuminate different relationships, connections, and networks between these communities [27–30].

Shells of marine molluscs have been one of the earliest materials and artefacts chosen to be used for adornment in human history. Early uses of shells as beads seem to be associated with the emergence of modern humans, appearing in Middle Palaeolithic and Middle Stone Age sites, dating between 140–70,000 years ago, in South Africa, North Africa and the Levant [31–36]. In the following Upper Palaeolithic period their frequency and diversity increased, especially in the Levant and Europe [37–39]. Specifically in the Levant, the Epipaleolithic cultures saw a dramatic increase in the quantity of shells in archaeological sites [40, 41], and in the Natufian, for the first time, a clear association with human burials, most notably of scaphopods [42–44].

The radical changes in human economy and social organization that followed the onset of sedentism and agriculture before and during the Neolithic period [45–49] also brought changes in the collection and use of shells by humans, and in the roles they performed within the social and economic lives of farming communities [50–53]. Starting during the 11^th^ millennium calBC [46: table 1, 54], the communities living in the Levant and adjacent regions, as a whole, went through a ’Neolithization’ process. Parts of the population, specifically those inhabiting the Mediterranean environmental zones, gradually adopted agriculturalist lifeways, established continuously growing permanent villages, and deepened the exploitation and dependency of the subsistence economy on domesticates. Contemporaneous populations in the desert regions maintained hunter-gatherer lifeways, yet with constant contact with the farming communities in the Mediterranean zones [46, 55, 56]. Rising population densities within permanent villages; craft specialization, non-kin social fractioning, and social differentiation; the intensification of long-distance exchange networks and tightening connections with remote populations–created new social contexts and relations between individuals and communities [23, 53, 57–60]. This evolving reality necessitated continuous development of personal and communal identity markers, or elaborate means for the "negotiation and reproduction of collective identities" [61].

Despite and alongside the above-mentioned differentiating circumstances between the desert and Mediterranean zones in the Levant, the Neolithic communities living in the region during the PPNB (mid-9^th^ to mid-7^th^ millennium calBC [62: table 1] were part of what is termed the ’Neolithic interaction sphere’, implying the existence of multiple different shared aspects of life, expressed though material culture [55], with varying degrees of local and regional diversity [61]. This entailed generally shared practices, such as the production of a repetitive array of chipped stone tools, using similar production methods [e.g. 63, 64], as well as shared social mechanisms, symbols, beliefs and world views [23, 53, 56]. The structure of social relationships holding this ‘interaction sphere’ together was described by Watkins [65] as ‘nested networks of communities’ [60: 160] involving the maintenance of local, regional and supra-regional networks. These terms imply a hierarchically tiered set of relationships, or widening circles of social connections and affiliations, in which people were "*formulating and expressing multi-layered identities …*” [65: 165]. Different elements of adornment are thus expected to be shared throughout the geographic range of the Neolithic interaction sphere, while specific aspects of it may be differentially expressed by various communities or groups of communities. Shell beads, and the regional distribution patterns of discrete shell bead types, may be used as proxies for the exploration of shared as well as unique PPNB adornment practices throughout the Levant.

**Fig 1:**
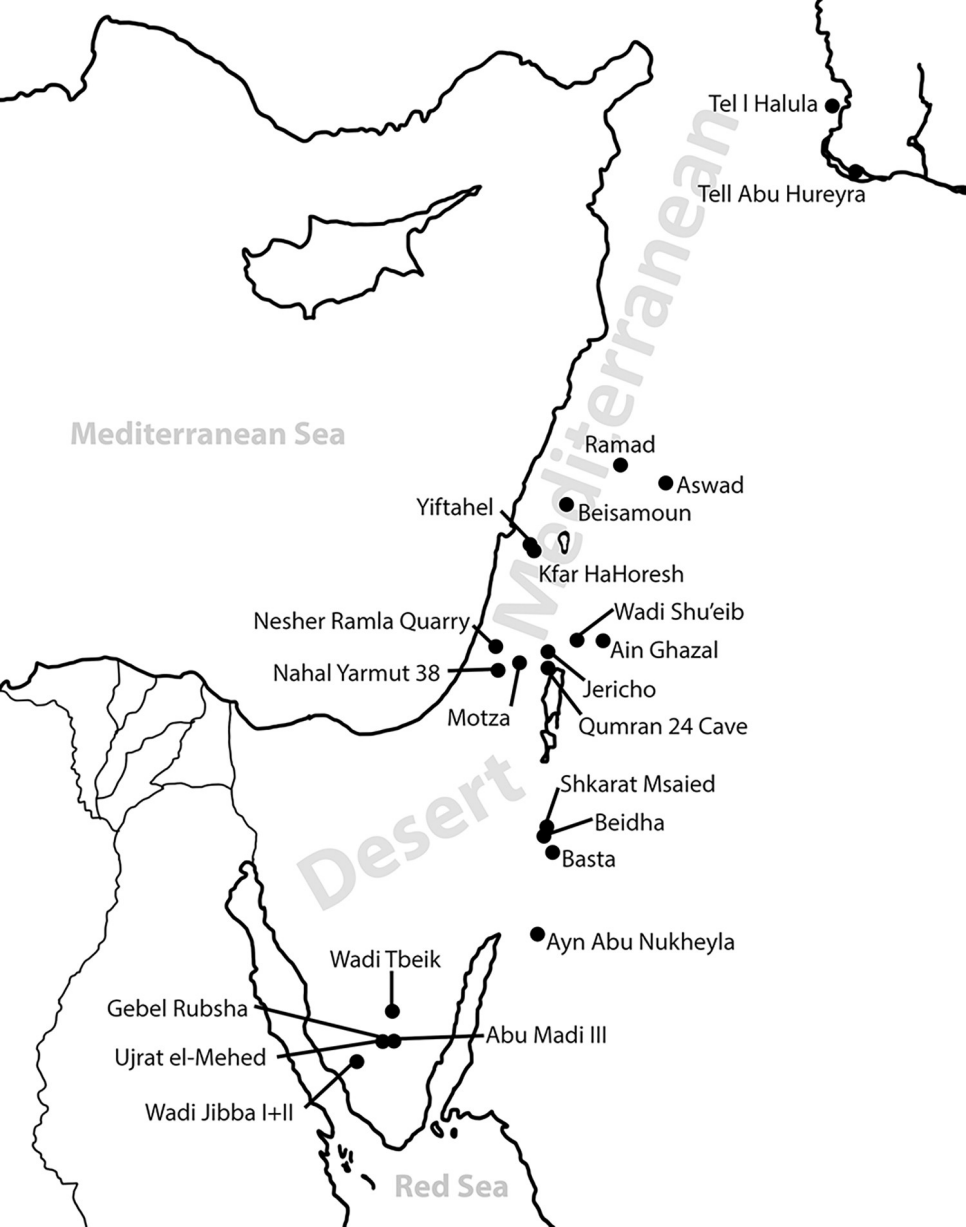
Map of sites mentioned in the text.

The cultic mortuary PPNB site of Kfar HaHoresh (KHH) [66–71], located in the western Nazareth hills of the Lower Galilee (Fig 1), serves as a focal point for the comparison of shell bead types found in different sites in the Levant. KHH has produced the largest shell assemblage among the PPNB sites in the Mediterranean climatic zone of the southern Levant, including over 2,000 marine shell specimens (NISP). Considering that the total excavated volume is ca. 400m^3^, shell density is extremely high compared to contemporaneous sites in the Mediterranean zone. Nearly a third of the shells were found naturally or artificially perforated or otherwise shaped to be used as beads.

Three stratigraphic phases, dated by radiocarbon and lithic techno-typological seriation, were recognized at KHH, representing the Early, Middle and Late PPNB phases, spanning the time period of ca. 8,600–7,250 calBC [68, 72, 73]. The site itself is almost hidden, nestled in a natural embayment delineated by a rock step, on the north-facing sloped bank of the uppermost reaches of a small wadi. There is virtually no arable land in the immediate vicinity of the site, and though the site itself has very limited visibility, the summit facing it has panoramic vistas of the entire Lower Galilee and adjacent regions [74: p. 197–203; 75].

A wide range of often unusual mortuary installations and practices were documented at the site, including single and multiple, primary and secondary burials, as well as several cached and/or plastered skulls [67, 76–78]. Grave goods of various types are often found, and some burials are associated with animal themes [72, 78, 79]. Evidence of feasting were documented in a series of pits containing *Bos* remains, indicating the consumption of several tons of meat in each session [72, 80]. Intensive pyrotechnical activity was also revealed at the site, concentrated on the production and use of large amounts of different types of lime plaster [69, 77, 81]. Extensive chipped stone working, in the form of multiple caches, refuse pits, manufacturing debris concentrations, and scattered lithic remains, amounting to hundreds of thousands of items, were also found [82–85]. Exotic artefacts and materials, such as obsidian [86], cinnabar [77], different greenstone minerals [87], or Red Sea Shells, were brought to the site from afar reflecting the participation of the people using KHH in wide-ranging, long-distance exchange networks, and their maintenance of direct and indirect relationships with remote communities. The architectural remains at the site changed through its occupation, from the use of a central monumental walled and lime-plastered podium in the earlier levels, to the use of multiple plastered surfaces, cists, installations, post-holes and combustion features, scattered in groups throughout the site, in the later levels [74: p. 71–74; 88].

Based on its location, and on the nature and contextual associations of the material cultural remains, the site of KHH has been interpreted as a cultic-mortuary communal gathering locale, serving the farming communities living in the nearby valleys. The shell bead assemblage collected at the site and the typological variety of shell beads found in it serve as the basis, and starting point, for the following analysis.

Relationships between Neolithic communities in the Levant have been traditionally studied through a variety of aspects such as settlement patterns, architecture, lithic typology and technology, treatment of the dead, and more [i.e. 55, 56, 60, 63, 65]. Here we use a different aspect of material culture–shell beads–to refine our ability to interpret such connections. In this paper we examine and define the variety of shell bead types found in different sites in the Levant, trace the geographic spread of the different types, and emphasize the scale and patterns of their distribution. Based on theories of social identity and the social roles of personal adornment, we hypothesize that the use of the same bead types by different communities represents expressions of social affiliation. Through multivariate analysis of the distribution patterns of different shell bead types we illuminate complex networks and community relationships. The results indicate that different shell bead types have different distributional scales, from pan-Levantine, through regional, to individual occurrences of unique types. These distribution patterns suggest that social relationships and identities were influenced by cultural affinities and not exclusively based on geographic proximity. We show, for example, that the communities using KHH had connections with the distant (>90km away) community of Jericho, as based on their shared use of special bead types and other shell related practices, unique only to them. The specific patterns of distribution of different bead types, and particularly their varying breadths, outline the hierarchically tiered structure of the Levantine Neolithic social identity.

## Methods

### Sites chosen for comparison

Shell beads from KHH and 23 additional sites were selected for comparison (S1 Table). Sites were selected depending on the accessibility of data concerning the shell bead assemblages; on the identification of a sufficient amount of shell beads from the site (sites with fewer than five beads were not included); and on their geographic location. The selection of sites provides a wide range, regionally encompassing sample, representing the geographical and climatic diversity of the Levant. The sites represent two general environmental zones found in the Levant–the Mediterranean and desert zones; as well as geographically distinct landscape units (’regions’) within these zones (Fig 1).

The sites selected for this analysis are not evenly dispersed across the study area and extensive regions in both the Mediterranean and the desert zones are not represented. This is due to multiple factors, such as research biases or limited shell publications, as well as possible true absences of PPNB sites or shell assemblages in some regions (i.e. [89] for Lebanon). Regarding the desert zone, it has been suggested that a distinct PPNB ’province’, encompassing the Negev highlands, western Negev and northern Sinai can be recognised [63: p. 143–150, fig. 7.3; 90], where marine molluscs are almost completely absent from sites [91]. At the few sites in that region where shells do appear, they may be considered as a curiosity [i.e. 92] or intrusive from earlier periods [i.e. 93, 94], and the assemblages were described as fragmentary [95] and anomalous [96]. Such sites are not a part of our sample.

We only considered shell beads assigned by the different sites’ excavators to the PPNB (8,500–6,400 calBC [59: table 1]), either from exclusively PPNB sites or, in multi-period sites, only those assigned to PPNB strata. The PPNB occupations at the sites examined here may not be entirely contemporaneous, yet all have at least a certain degree of chronological overlap [97] and share common PPNB material cultural characteristics. We recognize that there may be a diachronic aspect to the shell bead type variability found in the sample. However, the small number of shell bead assemblages available for analysis, along with other limitations regarding dating, stratigraphy, artefact collection, and reporting practices, currently prohibit further in-depth investigations of chronological change in shell bead type choices within the PPNB (a lacuna also mentioned in [52]). Further justification for the study of the entire PPNB as a uniform chronological entity regarding shell beads, comes from Perlès’ studies of the Greek Mesolithic phases at Franchthi Cave (contemporaneous to the Levantine PPNB) [99, 100]. Her studies demonstrate: a) pronounced continuity in different aspects of the shell assemblages throughout the sequence, and; b) no correlation between changes in sedimentary layers, lithics, and ornamental assemblages, leading to alternative phasing for each material aspect [98, 99: p. 9, 101–104, 197].

### Data collection

Taxonomic identification of shells from KHH, as well as other assemblages from the Mediterranean environmental zone (Beisamoun, Nahal Yarmut 38, Nesher-Ramla Quarry, Qumran Cave 24 and Yiftahel. S1 Table), was performed at The Steinhardt Museum of Natural History, Tel Aviv University, with comparison to the museum’s mollusc collection and different taxonomic guides [100–104]. The study of human manipulation of the shells was conducted through macro- and microscopic visual observations, adapted from previously established methodologies for technical and functional analyses of shell beads [such as 105–110], primarily regarding manufacture and use traces. The microscopic examinations were performed using a Zeiss Discovery.V8 Stereoscope (magnifications X10-X80) and a DinoLite digital microscope pro AD-413T (magnifications x20-x230). Images were photographed using an Axiocam 105 colour camera mounted on the stereoscope, analysed using compatible Zen software, and the in-built DinoLite camera, analysed using DinoCapture 2.0 software.

Shells from Ain Ghazal, Basta, Beidha, Jericho and Wadi Shu’eib were studied by D.S. Reese. Data regarding shells and shell bead types found in other sites was extracted from relevant publications (S1 Table). Taxonomic nomenclature is ever-changing; all out-dated taxonomic names, occasionally appearing in other reports, were unified according to the currently accepted version as it appears in WoRMS website [104].

### Defining shell bead types

Comparisons between assemblages are based on a shell bead typological list made up of 133 discrete shell bead types (S2 Table). Distinctions between the bead types are based on the shell taxa and general shape, the perforation technology, and the specific number and location of perforations on the shell body. The presence/absence of each shell bead type is indicated for every site in the sample, forming the basic dataset for the following analyses.

### Multivariate analysis

#### The use of binary data

Preliminary analyses of the shell assemblages included here were performed and reported by various researchers, at different times, and in variable detail. The archaeological material was collected through varying methods and extents of excavation, not always comparable in the degree and resolution of recovery. As shell beads tend to be small compared to other artefacts, they also tend to be under-represented in the recovered material, their numbers unrepresentative of their actual importance or frequency in the lives of the inhabitants of the site [as in 28, 30]. Additionally, the number of shell items made into each specific bead type was not always reported in the published analyses. The comparison of the occurrences of each bead type in the different sites is thus based on presence/absence data rather than specific counts.

#### Types of multivariate analyses used

A set of statistical tests aimed at exploring patterns of bead type distributions and connections between the sampled sites was employed. This analytical avenue and the use of the specific statistical and computational methods was inspired by the work of Rigaud et al. [28], regarding evidence for resistance to newly emerging Neolithic styles of personal adornment by indigenous hunter-gatherer societies in Europe, through the analysis of the types of personal adornment artefacts used; and that of Vanhaeren and d’Errico [30] on the identification of possible linguistic groups in the Upper Palaeolithic of Europe. Their methods are appropriate to deal with the research questions addressing the Levantine Neolithic. All computational procedures were performed using PAST 4.10 software [111], specifically designed for paleontological research.

Seriation, a data analysis technique that reorders objects into a unidimensional sequence [112], was used here to preliminarily explore different patterns that may arise from the data set. In the seriation analysis we included only those bead types that appear in more than one site. We performed the analysis in two forms, first constraining the order of sites to their north-south geographic order, in an attempt to identify geographic distribution patterns of the bead types, and then without constraining the order of sites, in order to examine the influence of bead type compositions on a similarity-based ordering of sites.

Non-metric multidimensional scaling (NMDS) is designed to represent ranked differences between data points, based on a distance matrix, by producing a gradient ordination of points [113, 114]. For the visualization of the data, it creates a scatter plot where each object is positioned closer to more similar objects and gradually farther away from gradually more different objects. It is used here to evaluate the ranked similarities between assemblages, meaning which sites have more, or less, similar shell bead assemblage compositions.

Clustering analyses are designed to group objects into clusters based on varying degrees of dissimilarities between objects [113, 114]. Neighbour-joining analysis is a method for hierarchical clustering analysis, used to help identify potential nested groupings, and specifically the shortest-branch clustering structure of a data set [115]. It is used here in an attempt to identify clustering patterns among the different sites, as based on the varying degrees of similarity between the compositions of their shell bead assemblages.

NMDS and Neighbour-joining analysis may be used to analyse binary data (presence/absence) with the mediation of designated distance matrices [113, 114]. We used the Dice coefficient for distance matrix pairwise calculations, as it places emphasis on instances of joint presence of bead types between sites, rather than on any incidence of absence, which, as mentioned above, may be circumstantial in the case of shell beads [as in 28].

One-Way ANOSIM is a non-parametric test of significance, examining the difference between groups as expressed by distance matrices. The test is based on comparing dissimilarities within-groups with dissimilarities between-groups [113, 114]. It was implemented here with the Dice index, in order to test the significance of presumed differences in shell bead compositions between regions.

### Highlighting the place of KHH within Levantine networks

Eventually, after examining the broad perspective through multivariate analyses, we concentrate on specific examples of common shell bead types and the distribution of their rarer variants, as they appear at KHH and other sites. This descriptive approach is used to demonstrate specific cross-regional connections within the pan-Levantine interaction sphere.

## Results

**Fig 2:**
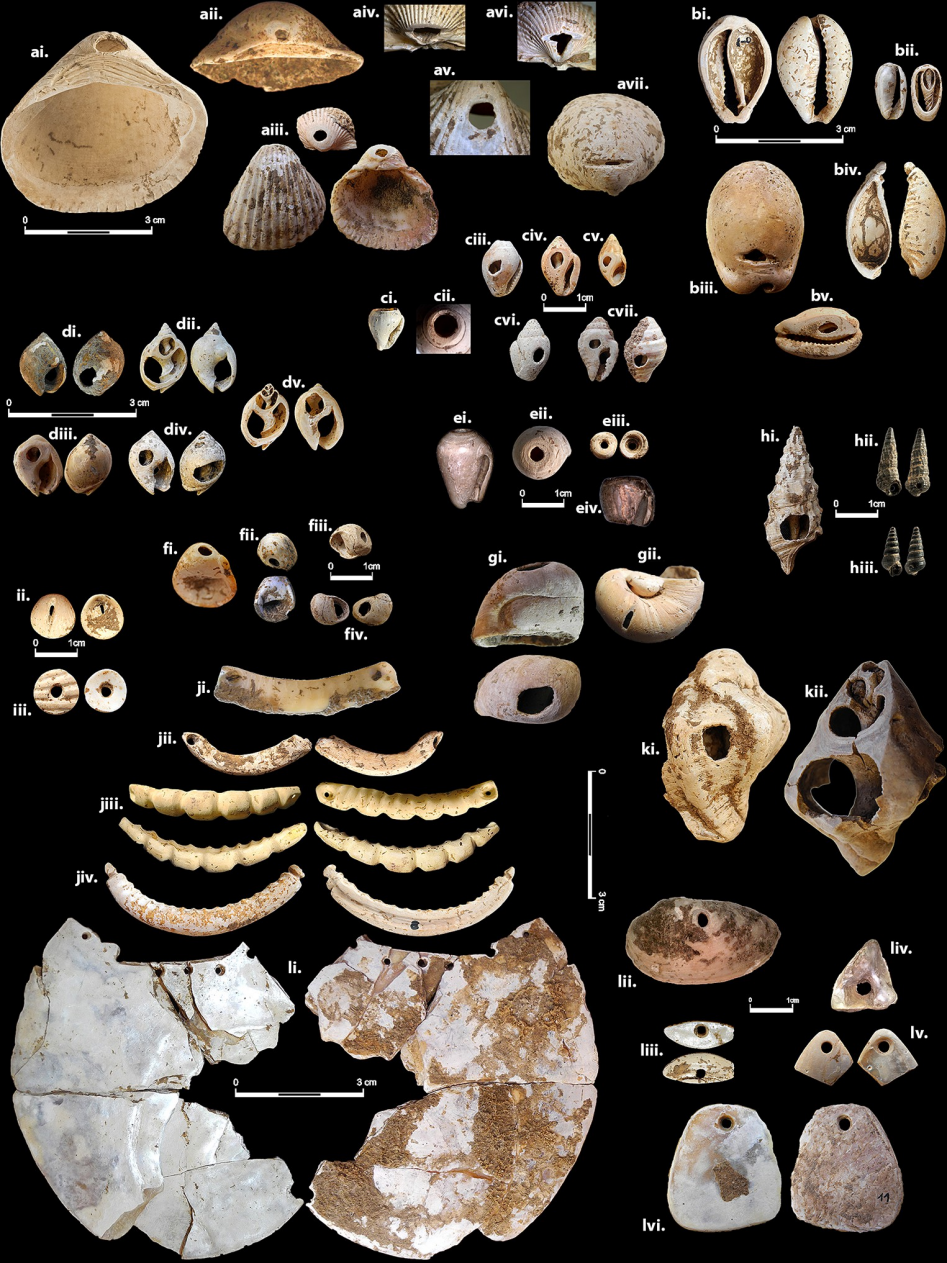
Examples of shell bead types found in the PPNB Levant. Notice distinction between types according to general shape/taxa, perforation location on shell body, and perforation technology. All images are scaled. All examples are from KHH unless otherwise stated below. Descriptions start with bead-type code as in S2 Table. ai. 1GGHBU. *Glycymeris* with a gouged/hammered/broken hole in umbo (Nesher-Ramla Quarry). aii. 1GGU. *Glycymeris* with ground hole in umbo. aiii. 1CGU. *Cerastoderma* with ground hole in umbo (top KHH, bottom Nahal Yarmuth 38). aiv. 1C-SGU. *Acanthocardia* with sawn and then ground perforation on umbo. av. 1CGD. *Cerastoderma* with ground hole on dome. avi. 1C+SU. *Cerstoderma* with cross-shaped slit in umbo. avii. 1GTSD *Glycymeris* with transverse slit on dome. bi. 2CDR. *Naria turdus* with removed dorsum. bii. 2VDRG. *Volvarina monilis*, dorsum removed by grinding. biii. 2CDCS1. *Luria lurida*, dorsum edge perforated by sawing (Nahal Yarmuth 38). biv. 2CDRLK1. *Naria spurca*, dorsum removed, columellar lip with anterior end shaped into a knob. bv. 2DCLG. *Luria lurida*, dorsum complete, ventral face of columellar lip perforated by grinding. ci. 4CASN. *Columbella*, apex/spire perforated by natural wear or breakage. cii. 4CASG. *Columbella*, apex/spire perforated by grinding. ciii. 4CFGHB. *Columbella*, front perforated by gouging/hammering or natural breakage. civ. 4CFG. *Columbella*, front perforated by grinding. cv. 4MFP. *Melanopsis*, front perforated by grinding. cvi. 4CBP. *Columbella*, back perforated. cvii. 4EMFB. *Engina mendicaria*, both front and back of shell perforated. di. 3TDP *Tritia gibbosula*, dorsum perforated (Beisamoun). dii. 3TDR. *Tritia gibbosula*, dorsum removed. diii. 3TCG. *Tritia gibbosula*, callus perforated by grinding (Nahal Yarmuth 38). div. 3TDPC. *Tritia gibbosula*, both dorsum and callus perforated. dv. 3TDRC. *Tritia gibbosula*, dorsum removed and callus perforated. ei. 5CASN. *Conus*, apex/spire perforated, unspecified technology. eii. 5CASG. *Conus*, apex/spire perforated by grinding. eiii. 5CTB. *Conus* ’top bead’. eiv. *Conus* ’barrel bead’. fi.-fii. 8NSW. *Nerita*, perforated on side of whorl opposite the aperture. fiii. 8TSW. *Theodoxus*, perforated on side of whorl opposite the aperture. fiv. 8TBS. *Theodoxus*, perforated on back behind aperture. gi. 9PBWGH. *Polinices mammilla*, gouged/hammered/broken hole on body whorl (Beisamoun). gii. 9NBWS. *Neverita Josephinia*, slit sawed on body whorl. hi. 6CFP. *Cerithium*, front perforated. hii.-hiii. 6PCBP. *Pirenella conica*, back perforated. ii. 11DB1S. *Cerastoderma* disc-bead, perforated by sawing. iii. 11DB1D. *Cerastoderma* disc bead, perforated by drilling. ji. 10CLDS. Cassid-lip, drilled holes, smooth body (Beisamoun). jii. 10CLDS. Cassid-lip, drilled holes, smooth body. jiii. 10CLDC. Cassid-lip, drilled holes, carved body. jiv. Cassid-lip, knob ends. ki. 7HBP. *Hexaplex trunculus*, back perforated. kii. 7HGIS+. *Hexaplex trunculus*, uniquely ground from apex to body whorl. li. 12MPRo3. *Pinctada margaritifera*, round pendant with multiple perforations. lii. 12MPV1. *Unio* perforated on the side of the dome (Beisamoun). liii. 12MPCr. *Unio*, crescent shaped pendant. liv. 12MPTri. *Unio*, triangular or rhomboid outline (Beisamoun). lv. 12MPTri. *Unio*, triangular or rhomboid outline. lvi. 12MPTrp. *Pinctada margaritifera*, trapezoidal shaped pendant.

A straightforward examination of the distribution of shell bead types (Fig 2, S2 Table) reveals that certain bead types are exceptionally common, appearing in most sites in the sample, while several occur in few sites, and others appear only once at a specific site. We noticed that different shell species that look similar, were treated in a similar way as in the case of Naticidae shells (including *Neverita*, *Polinices*, *Mammilla*, and *Natica/Notocochlis*); or with *Columbella*, *Pisania*, *Engina*, and *Euplica* shells (all belonging taxonomically to superfamily Buccinoidea and with a similar looking sculpture, despite being taxonomically attributed to different genera), as well as *Melanopsis* which is a somewhat similar looking freshwater shell (Fig 2 ciii-cviii or gi-gii). Results of the different analyses performed to explore this distribution pattern are detailed below.

**Fig 3:**
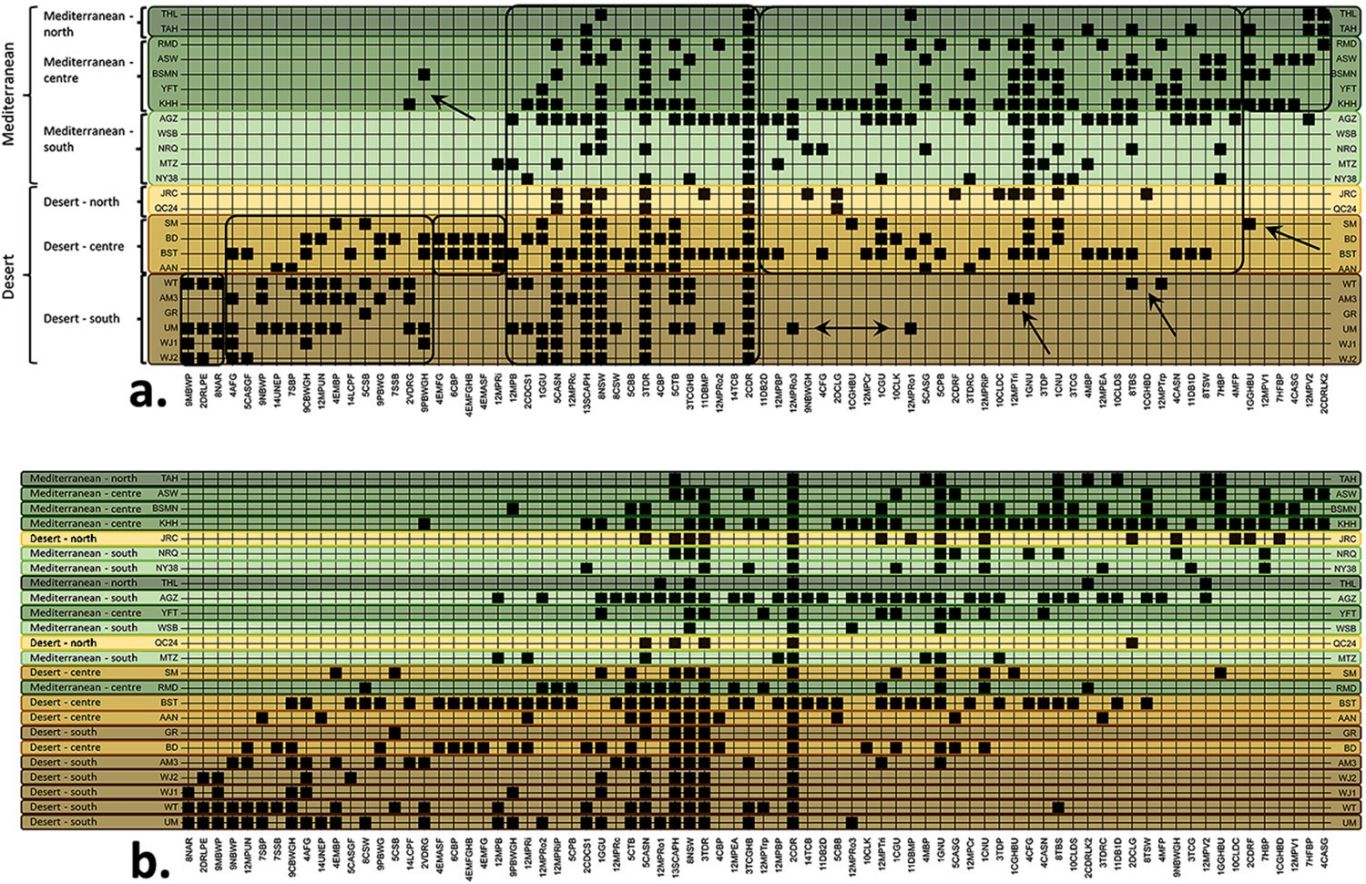
Seriation analysis. a. Sites constrained to their north-to-south distribution. Groups of shell bead types characterizing different regions or groups of regions are framed. Arrows point to examples of types found in sites beyond their typical geographic span. b. Sites unconstrained. Notice the changes in the order of the sites.

A large group of shell bead types may be considered as characterizing the entire sampled area (framed in the centre of Fig 3A). This group includes, for example, cowries (bead type code as appears in S2 Table: 2CDR) or *Tritia* shells (3TDR) with removed dorsa, *Nerita* shells perforated on the bulging side of the last whorl opposite the aperture (8NSW), complete *Conus* shells perforated through the apex and spire (5CASN), or Scaphopods (13SCAPH). An additional large group is shared by all regions except the Desert-south (shown to the right in Fig 3A), including for example naturally perforated *Glycymeris* (1GNU) and Cardiidae shells (1CNU) or ground Cardiidae (1CGU). An additional group characterizes the Desert-south and centre regions (shown to the left in Fig 3A), including, for example, *Clanculus* (9CBWGH) or *Polinices* (9PBWGH) beads. Smaller groups of shell bead types, represented on both edges of the seriation plot, typify only the Mediterranean-north and centre regions (on the far right), such as *Glycymeris* shells with a gouged hole in the umbo (1GGHBU), Unionidae shells double perforated in the hinge area (12MPV2), or cowrie columellar lips with knobs shaped at both ends (2CDRLK2), or only the desert-south region (on the far left), such as *Notocochlis* (previously *Natica*) beads (9MBWP) and *Nerita* aperture rings (8NAR). Some shell bead types are occasionally found beyond the limits of the region they typify (arrows point at some examples in Fig 3A). Seriation analysis performed while constraining the order of the sites to their north-to-south geographic distribution (Fig 3A) allows us to trace the geographic distribution of the shell bead types. According to the seriation results, it is possible to distinguish general groups of bead types characterizing each region or group of adjacent regions.

Removing the constraints on the geographic order of sites is also revealing (Fig 3B). It is apparent that the general Mediterranean-Desert distribution of sites is mostly maintained. However, within this general stable distribution, the sites in the Mediterranean-centre and south regions become intermixed, and the Desert-north site of Jericho is included among them, followed to a lesser extent by Qumran Cave 24 (Desert-north region) and Shkārat Msaied (Desert-centre region) (for the geographic location of sites see Fig 1).

**Fig 4:**
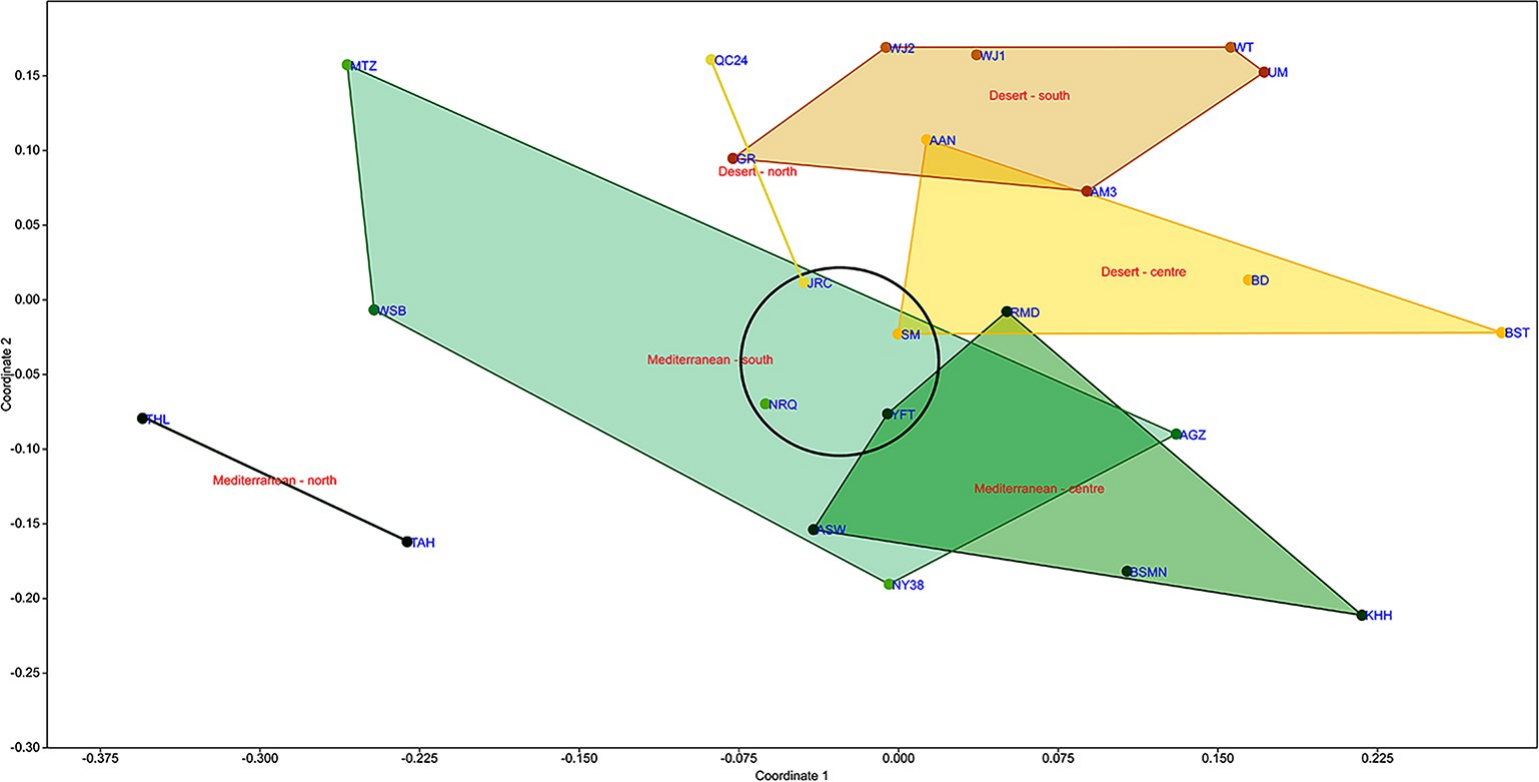
Non-Metric Multidimensional Scaling (NMDS). Encircled are sites from different regions that are closer to one another than to other sites in their respective regions.

The scatter plot produced by NMDS (Fig 4) similarly indicates a general distinction between sites in the different environmental zones and regions, with some overlap in the areas delineated by convex hulls. The most distinct are the sites in the Mediterranean-north region, while the largest overlap is found among the sites from the Mediterranean-centre and south regions. The Desert-north site of Jericho and the Desert-centre site of Shkārat Msaied are also found among the latter Mediterranean sites. NMDS is designed to display ranked distances between objects. Thus, the position of some sites on the scatter plot, closer to sites that are geographically remote from them, and further away from sites within their landscape unit (as defined here) or environmental zone (see examples encircled in Fig 4), is significant. These results should be interpreted cautiously, as the resulting stress value is quite high (0.2533), nearing a level indicating an arbitrary scatter.

**Fig 5:**
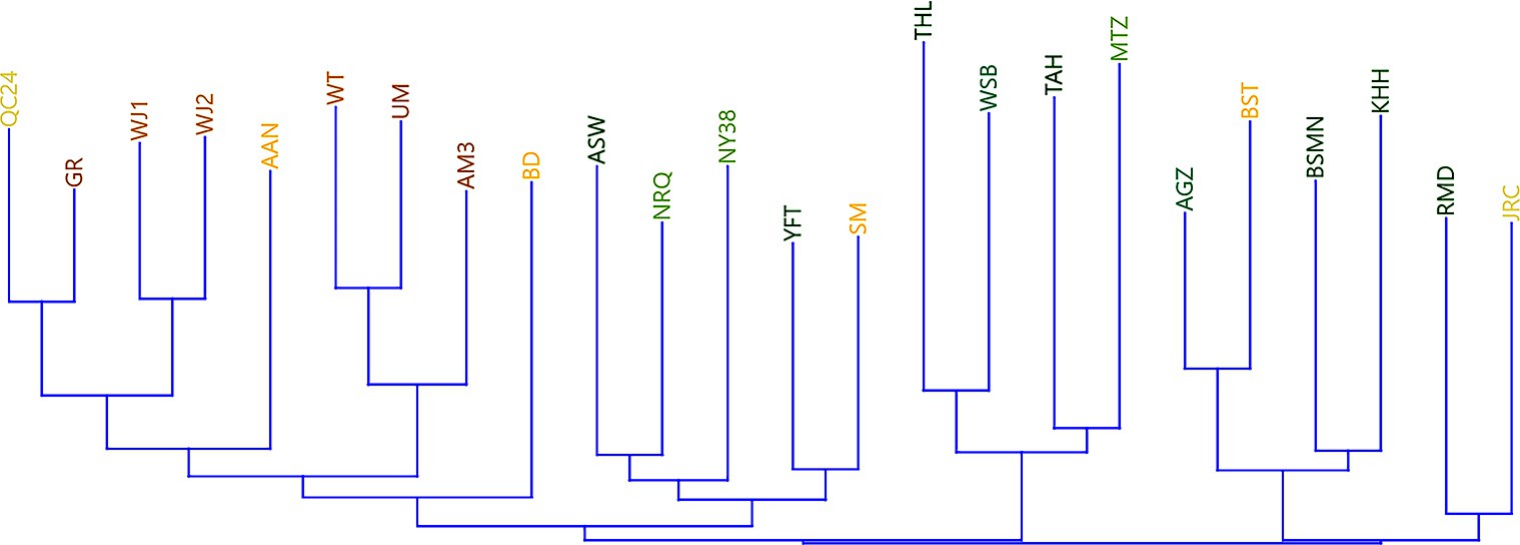
Neighbour-joining cluster analysis. Top cluster, with site names in yellow and brown, represents desert sites, while the bottom clusters, with site names in shades of green, are predominantly Mediterranean, yet do include several desert sites.

The dendrogram based on Neighbour-joining analysis (Fig 5) exhibits clusters that are generally, albeit loosely, correspondent to the geographic distribution of sites. One cluster is made up exclusively of desert sites, while the others include mainly sites from the Mediterranean environmental zone, with several sites from the Desert-north and centre regions intermixed among them (Shkārat Msaied, Basta, and Jericho).

The One-Way ANOSIM procedure showed that the overall dissimilarity between the environmental zones (Mediterranean and desert) is statistically significant (P = 0.0001), yet only slightly more dissimilar between zones than within each zone (R = 0.3616). A comparison between the landscape units displays a comparable overall dissimilarity (P = 0.0001; R = 0.4632). A pairwise contingency table, showing both *P* and *R* values produced by the comparison of the different regions (Table 1), indicates that the sites in the Desert-south region are the most significantly different from all other sites, and together with the sites in the Mediterranean-north region, are responsible for most dissimilarities between regions. The sites from the Mediterranean-centre and south regions, and those from the Desert-north region, show the least significant difference between them and the lowest dissimilarity values.

**Table 1:** One-way ANOSIM. P (= 0.0001) and R (= 0.4631) values for landscape units (’regions’).

	Mediterranean—north	Mediterranean—centre	Mediterranean—south	Desert—north	Desert—centre	Desert—south	
**Mediterranean—north**		0.0471	0.2373	0.343	0.0661	0.0361	*P-values*
**Mediterranean—centre**	0.6545		0.6374	0.2826	0.1591	0.0022	
**Mediterranean—south**	0.3	-0.046		0.3802	0.2503	0.0019	
**Desert—north**	1	0.2545	0.0636		0.1366	0.0348	
**Desert—centre**	0.8929	0.1688	0.1313	0.4286		0.0055	
**Desert—south**	0.9896	0.78	0.6773	0.5313	0.4762		* *
** **	*R-values*					* *	* *

**Notes:** orange shading indicates statistical significance (P<0.05). Blue shading indicates the greatest dissimilarities (R>0.5). Green shading indicates the greatest similarities and least significant differences.

## Discussion

The study of shell assemblages from 24 PPNB sites across the Levant, and the examination of the distribution patterns of 133 shell bead types forming these assemblages, reveal variable levels of connections between different components of the regional population. The examination of this complex network of relations enhances our interpretation of Neolithic society and identity across the Levant.

The distribution of items of personal adornment has been previously used to demonstrate populations’ social structure and identity, including social affiliation or disassociation, with particular reference to shell beads among other types of adornment [28, 30]. Following these conceptual frameworks, in this analysis we aim to outline the structure of Levantine Neolithic social identity, concentrating on the PPNB period, as it is expressed by the use of various shell bead types. The distribution of different shell bead types is used here as a reflection of the social networks active in the lives of the Neolithic people, influencing their adornment choices.

### Shell bead types as markers of identity: Definition, production, and use

Shell beads are usually attributed to composite forms of adornment. They constitute elements, or building blocks, used to create a wider visual effect [16, 116, 117]. The shape of each bead, the mode and direction in which they are strung, and the specific combinations in which they appear, may produce garments with different visual impacts [19, 106]. The desired visual outcome carries and transmits different culturally charged messages, which may pertain to different aspects of the wearer’s individual or collective identity [e.g. 12, 18–21]. The use of the same bead types is considered as an expression of mutual affiliation [27–30], as it implies the desire to embody and communicate similar messages concerning the individual’s or the community’s personal and social identities [13].

Stemming from this, the leading premise in the definition of shell bead types here, is that they were designed to have a recognizable and distinguishable look, either on their own or as part of composite adornment. Therefore, any aspect of a shell bead affecting its overall individual appearance should be taken into consideration. The identification and definition of the distinct shell bead types used in this analysis (S2 Table, Fig 2) was based on various observations, concentrating on the natural or manipulated bead shape, on manufacturing technology or lack thereof, and on perforation number and location.

**Fig 6:**
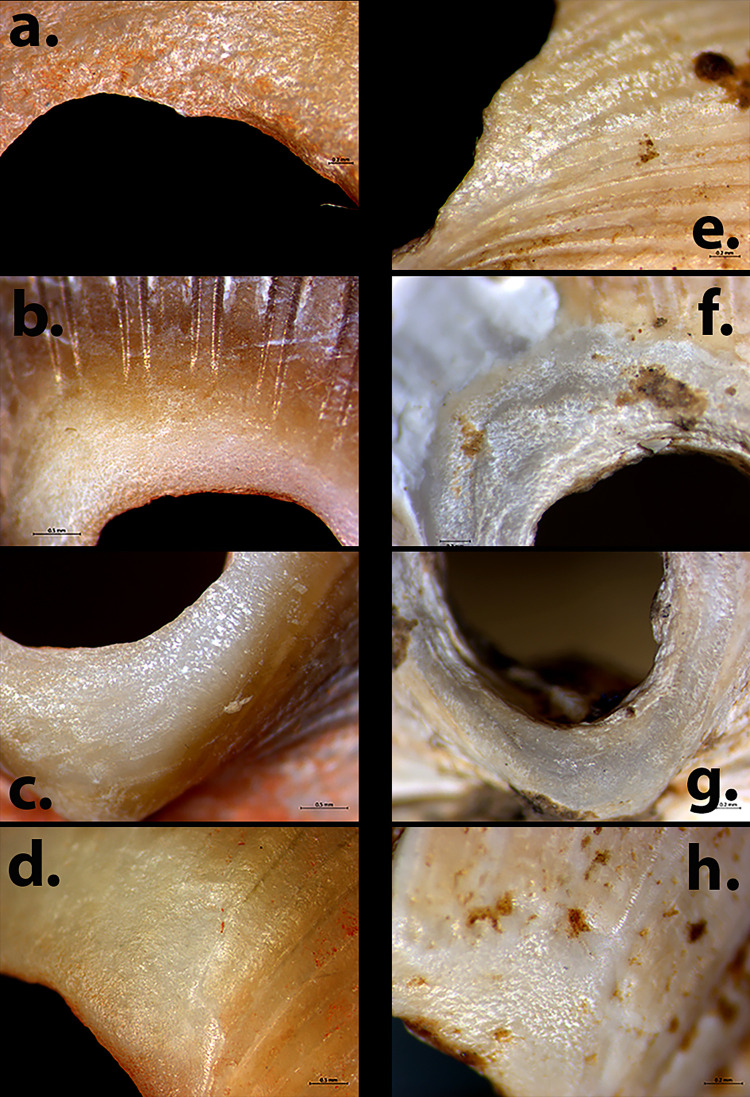
A comparison of rough (‘bumpy’) surface texture recognised on experimental and archaeological shell beads from Kfar HaHoresh. Experimental examples: a. *Cerastoderma* EX104. b-c. *Cerastoderma* EX108. d. *Cerastoderma* EX105. Archaeological examples: e. *Cerastoderma* #M1561 f-g. *Acanthocardia* #1343 h. *Acanthocardia* #544.

In the studied assemblages, artificially perforated and shaped shells were used alongside naturally perforated shells. Shells may become naturally perforated due to attrition of the empty shell by surf abrasion on the beach or sea-bottom, by trauma to the shell body, or different types of predation [118–120]. Naturally perforated shells have been continuously collected by humans, and used in their naturally perforated state, since the first introduction of shells into human material culture [31–36, 107, 110, 121–123]. Not all naturally perforated collected shells were necessarily used as beads. However, a specific type of wear, accumulated on experimentally strung and hung shells creating a rough (‘bumpy’) surface texture around strung perforations, was also identified on archaeological specimens (Fig 6), indicating that at least some naturally perforated shells were used as beads [124].

In other cases, artificial manufacturing of the shell beads was clearly apparent. Shells were turned into beads using various technologies, including gouging, hammering, grinding, and sawing [125, Technical terms after 126] (Fig 2). The manufacturing technology, like all technological actions, is a product of society, its culture, and habitual practices [e.g. 127]. It is thus an expression of particular cultural preferences and "ways-of-doing" related to shell beads and adornment in general. Shared technological practices, mutual ways of manipulating shells into beads, are thus also expressions of cultural and social ties between communities.

When turned into beads, shells may have been simply perforated, retaining their natural form, or manipulated into various shapes. The degree of manipulation exerted on the shell beads expresses a varying range of investment. When turned into beads, shells may have been simply perforated, retaining their natural form, or manipulated into various shapes. The degree of manipulation exerted on the shell beads expresses a varying range of investment, as seen for example, in the similar treatment of Naticidae shells or Buccinoidea shells (Fig 2 ciii-cviii or gi-gii). In both these groups of shells, communities from different parts of the Levant used similar looking shells from the nearest sources to produce a repetitive array of shell beads, essentially constituting ’material substitutions’ [116] for a desired bead shape. In other instances, locally available shells, such as freshwater *Theodoxus* shells found in several Mediterranean sites (8TSW), were used to imitate imported exotic beads such as the Red Sea *Nerita* common bead type (8NSW) (Fig 2 fi-fiii). Only this single type of *Nerita* bead had spread across the Levant during the PPNB. This typological singularity reflects strict conventions regarding recognizable bead types that may have been considered rare and precious. Those were possibly imbued with notions of prestige and privilege, and their imitation on locally available shells (e.g., the freshwater *Melanopsis* and *Theodoxus*) may have served different needs of the local society [128].

Despite having suggested instances of material substitution and imitation above, we are well aware of the possibility that Neolithic people did not perceive similarities between shell or bead types in the same way we do. Differences in perception were identified, for example, in the deviations between aboriginal Australian Gidjingali and Linnaean shell taxonomy [129: p. 48–56]. Moreover, peoples’ shell bead choices may have been driven by multiple symbolic, ontological, phenomenological, and/or practical factors, extending beyond mere stylistic considerations.

Other shells that were extensively manipulated, were sometimes fashioned into geometric forms, resembling beads made of various other materials during the same period. Beads made of minerals and stone [87, 130–132], and more rarely of plaster [133–135], wood [136], coral [137], or bone [138] (and particularly at Basta), were discovered in multiple PPNB sites in the Levant. They were occasionally shaped into forms also found made of shell, such as disc-beads, double-holed ovals, or rings/bangles [130: fig. 8, 10; 131: fig. 2, 8]. It is important to perceive the shell beads as part of a wide and diverse array of artefacts used for adornment; however, our current state of knowledge of non-shell ornaments in the region hinders a comparable study and their integration with shell ornaments.

Finally, after a shell bead has been produced, it was presumably used for adornment. The different uses and stringing modes of shell beads add multiple factors influencing their overall visuality. The location of the perforation on the shell allowed for different stringing directions, exposing different sides of the shell, or positioning it at different angles, and the number and size of perforations also influenced the specific hanging mode and display [105, 106, 139]. Another important factor of visual effect in adornment practices is colour. However, most archaeological shell specimens had lost their colour with time, and other references to colour (blackening, ochre) were only sporadically reported [140–142].

### The distribution of shell bead types and implications regarding regional relations

Previous studies have demonstrated patterns and relationships of Neolithic life. Based on lithic studies of bidirectional blade industries, Barzilai [63] defined the area included here in the Mediterranean-centre and south and Desert-north and centre regions, as one complex, made up of three facies and industries [63: p. 146, fig. 7.3], which generally correspond to the division between landscape units used here. Bar-Yosef and Bar-Yosef Mayer [56], considering multiple economic and cultural aspects, had concluded that the communities populating this area during the PPNB may be considered as one ‘tribal entity’ sharing multiple cultural markers of various aspects of life [56: fig. 8].

Our findings complement and expand upon these earlier suggestions. The seriation analysis demonstrates that there is a geographic orientation to the distribution of shell bead types across the Levant, with considerable overlap in the areas in which the bead types appear. Some bead types epitomize a region, or several regions, while others are widely shared throughout the study area. Shell bead types that were found to characterize a region represent the local adornment preferences and local social circles, while shell bead types that characterize several regions attest to the expansion of these ties. The appearance of multiple shell bead types in sites across the study area, as well as the occasional appearance of several geographically restricted shell bead types in sites far from their typical region/s, reflect the maintenance of long-distance networks and social relationships enabling widespread sharing of cultural markers throughout the Levant.

The seriation analysis has shown that sites that are related to one another, as based on the composition of their shell bead type assemblage, are not necessarily geographically adjacent. Sites in the Mediterranean-centre and south and the Desert-north regions are particularly intermixed, as is similarly expressed in the results of the Neighbour-Joining cluster analysis. While the bead type composition of Ramad (in the Damascus basin) resembles those of Shakrat Msaied and Basta (in Edom, ca. 340 km away to the south) more than that of nearby Aswad (Damascus basin, 40 km distant), as a rule, sites from the Mediterranean-north and the Desert-south regions remain more stable in their position at the ends of the seriation continuum.

The results of NMDS indicate that the different regions are fairly distinct, especially the Mediterranean-north region, and display separable shell bead type compositions. However, a considerable degree of overlap is apparent, particularly among the sites in the Mediterranean-centre and south regions, including parts of the Desert-north and centre regions. This fits well with the results of the one-way ANOSIM that found that the regions at the margins of the study area, namely the Desert-south and Mediterranean-north regions, display the most dissimilarity and contribute most to the significance of these differences, while the Mediterranean-centre and south and the Desert-north regions are the most alike. These results also correspond well with the results of the unconstrained seriation analysis presented above, together reflecting gradient levels of shell bead type sharing throughout the Levantine interaction sphere. The distinction of shell bead assemblages from the margins of the study area reflects the existence of local adornment preferences, as well as proximity to the shell sources that are common within each region and generally not inter-regionally shared. The overlapping or intermixing of assemblages from the centre of the study area reflect more shell bead type sharing that reflect intensive interactions among these sites and regions.

### Relationships between sites unrelated to geographic proximity or shared environmental conditions

In several instances, identified in both the NMDS and Neighbour-joining, as well as in the seriation analyses, assemblages from remote sites were found to be more similar than those of nearby sites or from other sites in the same region. This highlights the intra-regional variability in shell bead choices and indicates that adornment preferences were not strictly limited to geography. The observed similarities between remote sites represent the establishment of relationships and affiliations between communities (or individuals), unrelated to their geographic proximity.

Geographic proximity has been regarded in the past as the basis for social identification. De Swaan [143], for example, described it through concepts of widening circles of affiliation. He suggested that the first expansion of identification beyond kinship lines occurred with the onset of agriculture and Neolithic lifeways and was based on proximity–identification with the village and community one lives in and with neighbouring villages. Rigaud et al. [28] has, however, recently found only slight correlation between Mesolithic/Early Neolithic bead-type diversity and geographic distances across Europe, with surprising similarities and associations between geographic extremities. Our results similarly show that the Neolithization process in the Levant not only did not restrict the formation of circles of identification to the bounds of geographic proximity, but probably even fostered their expansion through the establishment and maintenance of long distance relationships. Social ties and networks, based on a varied myriad of underlining motivations, thus spanned far beyond the constraints of geographic proximity and relationships strong enough to impact adornment choices, and were maintained during the Neolithic across great distances throughout the Levant.

Differences in environmental conditions between the Mediterranean and desert zones were also bridged, especially in bordering regions. The most obvious case is that of Jericho, which according to the results of all analyses, displays a Mediterranean-style shell bead assemblage, rather than a desert one. Despite being situated in a desert environment, characterized by year-round hot and extremely dry climatic conditions caused by the rain shadow effect and a barren petrous desert landscape [144, 145], comparable to other desert landscape units included in this analysis, Jericho seems to culturally belong to the Mediterranean milieu. This Mediterranean affiliation has been recognized in chipped stone production technologies and typological stylistic preferences as well [64: p. 246, 260, figs. 8.5–8.6; 146: p. 702–706]. The people living in Jericho thus seem to have had closer cultural and social connections to communities living in the adjacent Mediterranean environmental zone to the west and east across the Rift Valley than to those living in the desert, despite their location within a desert environment.

### Bases for maintaining long-distance relationships

The statistical analyses used to examine the distribution patterns of shell bead types indicate that not all social circles and affiliations are based on geographic proximity and may be related to various systems and networks operating in the greater region. As fractions of the population were continuously moving across the landscape, even after the wide-scale adoption of sedentary life, and regularly interacting on different levels [56, 59, 65], social circles may be based on multiple contextual connections other than geographic proximity.

For example, different systems of resource exploitation may drive interactions between geographically distinct groups. The cyclical movement between environmental zones driven by the seasonal availability of resources [147], may generate interactions between remote communities, specifically regarding the relationships found between Mediterranean communities, and those from the Judean desert, such as Jericho. Such seasonal, or climatic and environmentally based, movements have been proposed to explain earlier hunter-gatherer Harifian [148] and later farming Chalcolithic [149] settlement patterns, as well as contemporaneous and later Neolithic systems in the desert regions of the Southern Levant [56, 150, 151]. The availability and exploitation of other, non-seasonal, resources may also drive the establishment of relationships and exchange networks between remote communities. Dead-Sea asphalt was identified in Mediterranean sites such as at Nahal Yarmut 38 [152], and possibly at KHH [153] (though see [77, 154]), the acquisition of which presumably involved the participation of local communities in both areas.

Obsidian exchange networks, active in the region from as early as the Epipalaeolithic Natufian period [155], facilitated the trade in obsidian during the PPNB, from its Anatolian origins, through the Southern Levant, to the Negev desert [156–159]. The model of obsidian exchange and distribution, originally described in ‘down-the-line’ terms [160, 161], has since been understood as more sophisticated, involving the exchange of goods between ‘centres’ [162]. Recent analyses based on Network Theory explain PPNB obsidian distribution as deriving from exchange between ‘nodes’ (sites, villages) connected both to their immediate ‘neighbourhood’ (nearest surrounding sites) as well as to distant ‘nodes’ (more remote sites), creating distant exchange links [163–165]. This type of complex regional exchange network, made up of varying connections between near and distant communities, found its expression also in the choice of shell bead types.

Furthermore, marriage arrangements and genealogical ties likely connected between remote communities. Familial and biological relationships among individuals buried at KHH were investigated based on indicative tooth morphologies [166], showing the presence of a biological cluster of females and sub-adults within the general heterogeneous population. Males buried at the site were found to be biologically unrelated to the cluster, possibly suggesting matrilineal exogamous practices based on the introduction of male individuals from different groups into the community. Additionally, a Neolithic mtDNA study [167] has shown a common genetic pool for the PPNB populations at Tell Halula (Mediterranean-north here) and Tell Ramad (Mediterranean-centre here), living more than 350 km apart. Such mating practices and the maintenance of genealogical relationships between distant communities may have facilitated the use of shared identity markers such as specific shell bead types. Thus, trade and exchange networks, genealogical connections, or political alliances serving various interests, may have been formed and maintained between geographically distinct communities, and these affiliations, constituting practical social circles, may have motivated the shared use of different shell bead types.

### Test-cases: Tracing networks created by the distribution of cowrie beads and cassid-lips

**Fig 7:**
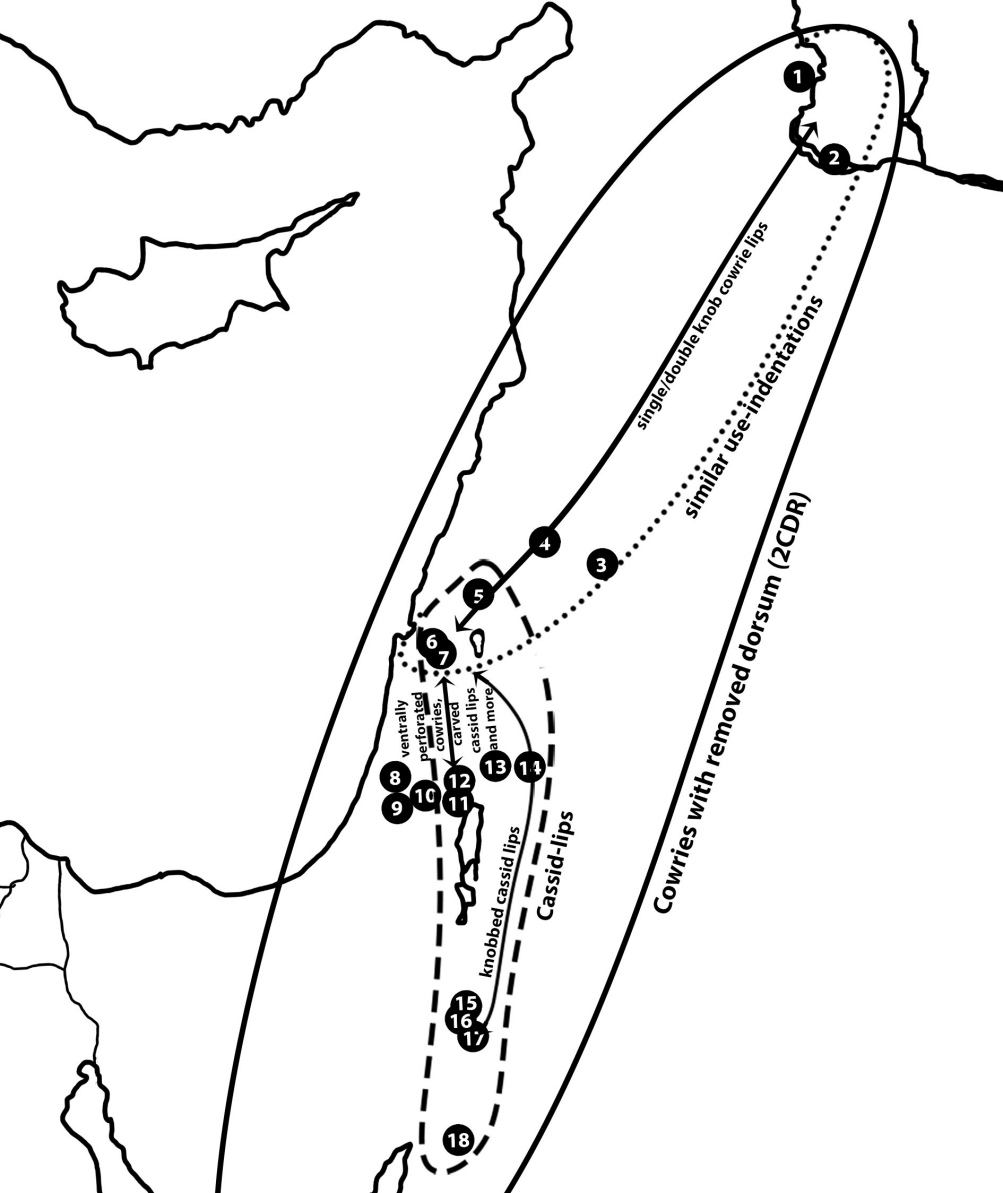
A schematic representation of networks related to KHH, based on the distribution of different types of cowrie beads and cassid-lips. List of sites: 1. Tell Halula. 2. Tell Abu Hureyra. 3. Ramad. 4. Tell Aswad. 5. Beisamoun. 6. Yiftahel. 7. Kfar HaHoresh. 8. Nesher-Ramla Quarry. 9. Nahal Yarmuth 38. 10. Motza. 11. Jericho. 12. Qumran 24 Cave. 13. Wadi Shue’ib. 14. Ain Ghazal. 15. Shkārat Msaied. 16. Beidha. 17. Basta. 18. Ayn Abū Nukhayla.

As mentioned above, some of the shell bead types that appear in many of the PPNB sites in the Levant are widely shared among communities on a large scale and represent the pan-Levantine cultural interaction sphere. Cowries with a removed dorsum (2CDR) (Fig 2bi) are one such type. They are very common in some sites (such as at Tell Halula [168]) and exceedingly rare in others (less than 2% of the assemblages from Yiftahel [169], Nesher-Ramla Quarry [142], or KHH). Nevertheless, they appear in all sites in the sample and are a characteristic feature of the PPNB Levant (Fig 7).

**Fig 8:**
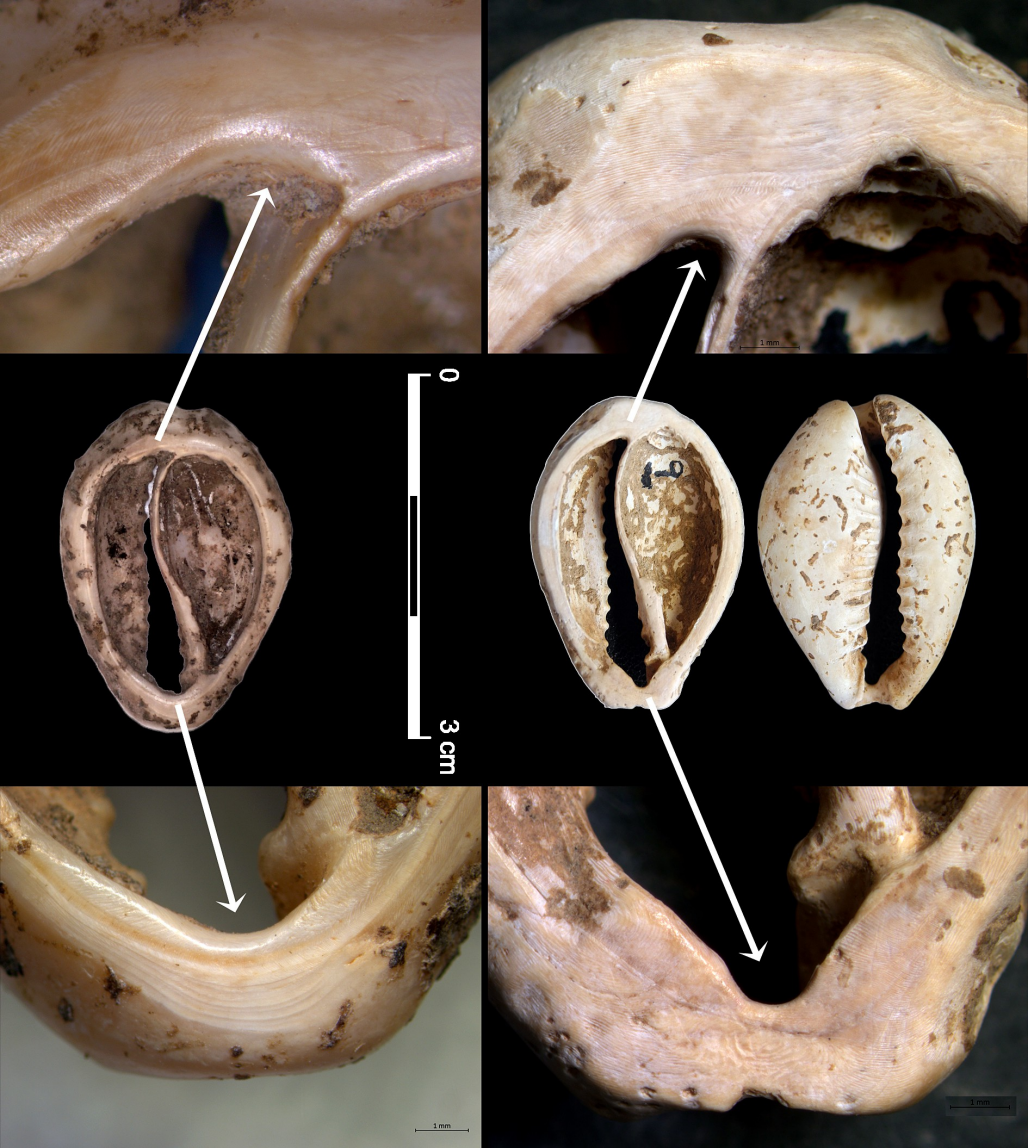
Use-indentations on cowrie beads. Left: *Naria turdus* #340 from Yiftahel. Right: *Naria turdus* #M830 from KHH.

This shell bead type may be used and strung in different ways. Use-indentations on the extremities of the dorsum removal-line are indications of stringing or sewing through either one or both ends of the aperture and are repeatedly found on specimens from KHH, Yiftahel (Fig 8), Aswad [139: fig. 9], Tell Halula [168: fig. 6], and Tell Abu Hureyra [170: fig. 3]. *In situ* finds at Tell Halula, where such cowrie beads were found still arranged in rows or chains, placed around pelvises and heads of interred individuals [168: fig 2c, e, f], corroborate this stringing-mode interpretation. The repeated use of the same bead type, stringing mode, and bead arrangement, suggests that the desired visual effect, and the messages conveyed by it, were widely shared, at least throughout the Mediterranean-centre and north regions (Fig 7).

Additional bead types were made on cowrie shells, yet these appear in fewer sites. For example, a different cowrie bead type was made of only the inner lip of a cowrie with a removed dorsum, featuring a circular groove at each end, creating knobs presumably designed to facilitate suspension by tying (2CDRLK2). Of all the sites in our sample, this bead type was only found at Tell Halula [168: fig. 2j], Tell Abu Hureyra [170: fig. 3] and Ramad [171: p. 173]. Yet a further variant, with a knob only at one end of the lip (2CDRLK1) (Fig 2biv), thus suggesting a different hanging posture and different visual effect, was found at KHH. Despite being different bead types, the shell type, a cowrie, and the idea of creating knobs to facilitate stringing, are shared.

This technological and stylistic idea is further found on ’cassid-lips’. ’Cassid-lips’ are the naturally detached outer lips of *Semicassis* shells, used to create concave crescent pendants [172]. ’Cassid-lips’ with knobbed ends (10CLK), delineated by grooves or notches, were found at KHH (Fig 2jiv), Ain Ghazal, and Beidha. Other variants, alternatively perforated by drilled holes with smooth or carved bodies (10CLDS/10CLDC), were found at Beisamoun (Fig 2ji), KHH (Fig 2jii-jiii), Ain Ghazal, Basta, and Jericho [173: fig. 2n; 174: fig. 253.5] (Fig 7). Besides this bead type, additional rare shell bead types seem to be shared exclusively, or almost exclusively, by KHH and Jericho. Such are cowries with removed dorsa in which both shell extremities are flattened (2CDRF) and ’cassid-lips’ with geometric carvings on the body (10CLDC) (Fig 7).

A different variant of cowrie beads has a complete dorsum and a ground hole in the centre of the ventral face of the inner lip (2DCLG) (Fig 2bv). Such beads were found only at KHH in the Galilee (Mediterranean-centre here), and at Jericho [173: fig. 2b, c] and Qumran Cave 24 [175: fig. 11d] in the Judean desert (Desert-north here). Other variants perforated on the ventral face of the inner lip were found at Aswad [139: fig. 2e, i, j], yet in each case they deviate, either in the technology of drilled holes, in the location of the hole along the lip, or by the dorsum being removed or also perforated.

### Widening circles of affiliation and the hierarchical nature of Neolithic identity

This outlined web of connections between communities (Fig 7), reflected by the sharing of specific taxonomic, technological, and typological choices, as well as stringing preferences, is an expression of the social circles and networks in which these communities were interacting. The shared rare bead types illuminate segments of the network that are particularly bound and operate within the greater regional network, represented by the shared common bead types. The rare shared occurrences tie together specific or individual communities, thus expressing narrower social circles that exist concurrently within the wider Neolithic milieu.

Additional connections between communities in the Galilee and the Judean desert are also found in other aspects of shell use, for example, in relation to plastered skulls. Skull removal practices are known from as early as the Epipaleolithic Natufian period in the region, and during the PPNB are found across the Levant and beyond [176–180: Fig 1]. Plastered skulls appear in some of the sites in the region [77, 180: fig. 1, 181, 182], representing a variation in skull-treatment practices, shared by a more closely related social and cultural circle of communities. The use of shells as part of the skull-plastering practice, embedded in the eye sockets of plastered skulls, is very rare, and appears only at Yiftahel [169, 183, 184] and at Jericho [180, 185–187]. Shells were also found embedded in the eye slots of a plaster head-statue from Jericho [180: pl. LIII; 188: fig. 19] and in the eye sockets of an ochred skull from Çatalhöyük (>600 km distant) (but not on the plastered skulls from Kfar HaHoresh) [189, 190], incidences that similarly represent a variation of a specific practice. It is thus evident that as the different variants of skull-treatment practices become more detailed and particular, they are shared by tighter and tighter social circles, within the wider cultural interaction sphere existing in the region during the PPNB. The different distributions of these variants are further examples expressing the different scales of social circles active in the lives of the Neolithic people, reflecting the hierarchical structure of PPNB social identity.

## Conclusions

People associate themselves to multiple social and cultural circles, creating affiliations that constitute fundamental aspects of personal and social identity. The use of personal adornment plays a part in both the embodiment and the construction of these aspects of identity, as well as in their external demonstration and communication.

In order to effectively communicate the desired messages, items of personal adornment must create a specific, deliberate, and recognizable visual effect. The shape, appearance, mode of manipulation, manner of use, posture, and more, of every item of personal adornment used, has crucial roles in the creation of the desired look [15, 16, 25, 116]. The shared uses of repetitive arrays of items of personal adornment, presumably designed for the creation of the same visual effects and the portrayal of similar messages, expresses mutual affiliation and an association with the same social and cultural circles.

The study of different aspects of social identity and social connections through the use of items of personal adornment must thus include a clear definition of types and systematic recording of the distribution of such items. The combination of detailed descriptions of individual micro-relations, along with the use of advanced statistical tools such as multivariate analysis that provide a broad macro-overview, enables a holistic perspective on the subject.

In this analysis we examined shell beads, as representative items of personal adornment, from PPNB sites across the Levant. The PPNB period in the Levant is a time of fundamental shifts in human lifeways, revolving around heightened sedentism and the adoption of agricultural practices, yet involving multifaceted reconfigurations in all aspects of life. Social connections, alliances, and networks were essential factors in these evolving adaptations, shaping the emerging Neolithic identities. Here we considered the various shell bead appearances as a basis for the definition of shell bead types and examined the distribution of these types across the Levant. We referred to the shared use of different shell bead types among communities from various sites as indications of shared cultural markers and thus reflections of mutual affiliation and the existence of social connections. Through the examination of different shell bead type distribution patterns, we suggest that Neolithic communities maintained different levels of connections with other communities, creating widening social networks. Some relationships were based on geographic proximity, creating local adornment styles. Our results, from across the Levant, reaffirm previous suggestions for the existence of cultural territories [63], or ‘tribal entities’ [56], tying together different parts of the Neolithic Levantine population. Other relationships involved connections between remote sites and were presumably based on various alternative motivations, such as trade, seasonal exploitation of resources, genealogical connections, and more.

Shell beads found at the cultic-mortuary aggregation site of KHH reveal multiple connections between the communities using the site and other communities in the Levant. As seen in the multivariate analyses, they are first and foremost linked to the general Mediterranean “style”, and particularly to the central and southern areas of the Mediterranean environmental zone. However, shared innovations in stringing-mode design (‘knobs’) and particular manners of shell bead position while stringing (location of use-indentations), reflect connections to communities living far to the north of the Galilee. At the same time, shared unique bead types (specific cowrie and cassid-lip variants) and other shell related practices (the use of shells in the eyes of plastered skulls) indicate particularly close relations between some communities in the Galilee and those in the northern Judean desert, specifically at Jericho. It is thus apparent that local, regional, and pan-regional social networks were at play in the lives and identities of the Neolithic inhabitants of the Levant.

To conclude, within the huge cultural and social ’supra-group’ constituting the ’PPNB interaction sphere’, people presumably affiliated themselves with multiple narrower social circles, each contributing to the constitution of the individual’s social identity and feelings of social assimilation and differentiation [23]. These affiliations presumably received an external expression in the form of personal adornment. By tracing the distribution of the different bead types found in the Levant, and by distinguishing between widely spread, exclusively shared, or uniquely occurring bead types, we may outline a hierarchically structured set of connections and relationships between communities in the region. Specific bead type distributions indicate that these connections were not simply dictated by geographic proximity or constrained by it, and may have been based on extended kin relations, moieties, gender, age, social status, as well as economic or political interests, trade relations and so on. In this paper we have discussed how nested networks [65], or hierarchically widening circles of social and cultural affiliations, which defined and constructed the PPNB social identity, were expressed through the use of shell beads as items of personal adornment, during the pivotal time of the PPNB in the Levant.

## Supporting information

S1 Table PPNB sites in the Levant used for the construction of the typological list and for comparison. (DOCX)

S2 Table Shell bead typological list and the occurrence of each bead type in the sampled sites. (DOCX)

## References

[1] Hogg MA, Abrams D. Social identifications: a social psychology of intergroup relations and group processes. London: Routledge; 1988.

[2] Tajfel H. Social categorization, social identity and social comparisons. In: Tajfel H, editor. Differentiation between social groups. London: Academic Press; 1978. pp. 61–76.

[3] Tajfel H, Turner JC. An integrative theory of intergroup conflict. In: Austin WG, Worchel S. editors. The social psychology of intergroup relations. Brooks/Cole: Monterey. 1979. pp. 33–47.

[4] Turner JC, Hogg MA, Oakes PJ, Reicher SD, Wetherell MS. Rediscovering the social group: a self-categorization theory. New York: Blackwell; 1987.

[5] Brewer MB, Gardner W. Who is this "We"? Levels of collective identity and self-representations. J Pers Soc Psychol. 1996;71(1): 83–93. doi: 10.1037/0022-3514.71.1.83

[6] Hornsey MJ. Social identity theory and self-categorization theory: A historical review. Soc Personal Psychol Compass. 2008;2/1: 204–222. doi: 10.1111/j.1751-9004.2007.00066.x

[7] Trepte S, Loy LS. Social identity theory and self-categorization theory. In: Rössler P, Hoffner CA, Zoonen L, editors. The International Encyclopedia of Media Effects. New York: Wiley; 2017. Pp. 1–13. doi: 10.1002/9781118783764.wbieme0088

[8] Scheepers DT, Ellemers N. Social identity theory. In: Sassenberg K, Vliek MLW, editors. Social psychology in action: evidence‐based interventions from theory to practice. New York: Springer; 2019. pp. 129–143. doi: 10.1007/978-3-030-13788-5_9

[9] Stets JE, Burke PJ. Identity theory and social identity theory. Soc Psychol Q. 2000;63(3): 224–237. doi: 10.2307/2695870

[10] Gaither SE. The multiplicity of belonging: pushing identity research beyond binary thinking. Self Identity. 2018;17(4): 443–454. doi: 10.1080/15298868.2017.1412343

[11] Hodder I. The Distribution of Material Culture Items in the Baringo District, Western Kenya. New Series. 1977;12(2): 239–269.

[12] Wobst HM. Stylistic Behavior and Information Exchange. In: Cleland CE, editor. For the director: research in honor of J. B. Griffin. Ann Arbor: University of Michigan, Museum of Anthropology; 1977. pp. 317–342.

[13] Fisher G, Loren DD. Embodying identity in archaeology. Introduction. Camb Archaeol J. Special Selection 2003;13(2): 225–230. doi: 10.1017/S0959774303210143

[14] Joyce RA. Archaeology of the body. Annu Rev Anthropol. 2005;34: 139–158. doi: 10.1146/annurev.anthro.33.070203.143729

[15] Kuhn SL. Signaling theory and technologies of communication in the Paleolithic. Biol Theory. 2014;9: 42–50. doi: 10.1007/s13752-013-0156-5

[16] Kuhn SL, Stiner MC. Body ornamentation as information technology: towards an understanding of the significance of early beads. In: Mellars P, Boyle K, Bar-Yosef O, Stringer C, editors. Rethinking the human revolution. Cambridge: McDonald Institute for Archaeological Research; 2007. pp. 45–54.

[17] Vanhaeren M. Speaking with beads: the evolutionary significance of personal ornaments. In: d’Errico F, Blackwell L, editors. From tools to symbols: from early hominids to modern humans. Johannesburg: Witwatersrand University Press; 2005. pp. 525–553.

[18] Mulleta AD, Muda K. Deciphering meanings embedded in the cultural ornaments of Guji Oromo women of Southern Ethiopia. Heliyon. 2021;7(4): e06774. doi: 10.1016/j.heliyon.2021.e06774 33912725 PMC8066376

[19] Wiessner P. Reconsidering the behavioral basis for style: a case study among the Kalahari San. J Anthropol Archaeol. 1984;3: 190–234. doi: 10.1016/0278-4165(84)90002-3

[20] Wiessner P. Style and changing relations between the individual and society. In: Hodder I, editor. The meaning of things. London: Harper-Collins Academic; 1989. pp. 57–61.

[21] Wiessner P. Seeking guidelines through an evolutionary approach: style revisited among the! Kung San (Ju/’hoansi) of the 1990s. Arch P Amer Ant Asso. 1997;7: 157–176. doi: 10.1525/ap3a.1997.7.1.157

[22] Wobst HM. Style in archaeology or archaeologists in style. In: Chilton ES, editor. Material meanings. Salt Lake City: University of Utah Press; 1999. pp. 118–132.

[23] Benz M, Gebel HGK, Watkins T. The construction of Neolithic corporate identities. Introduction. In: Benz M, Gebel HGK, Watkins T, editors. Neolithic corporate identities. Studies in early Near Eastern production, subsistence, and environment 20. Berlin: exoriente; 2017. pp. 1–9.

[24] Iliopoulos A. Early body ornamentation as Ego-culture: tracing the co-evolution of aesthetic ideals and cultural identity. Semiotica. 2020;232: 187–233. doi: 10.1515/sem-2019-0073

[25] Sterelny K, Hiscock P. Symbols, signals, and the archaeological record. Biol Theory. 2014;9: 1–3. doi: 10.1007/s13752-013-0154-7

[26] Schick T. Cordage, basketry and fabrics. In: Bar-Yosef O, Alon D, editors. Nahal Hemar Cave. ‘Atiqot. 1988;18: 31–43.

[27] Borić D, Cristiani E. Taking beads seriously: prehistoric forager ornamental traditions in Southeastern Europe. PaleoAnthropoly. Special Issue: Personal ornaments in early prehistory 2019: 208–239. doi: 10.4207/PA.2019.ART132

[28] Rigaud S, d’Errico F, Vanhaeren M. Ornaments reveal resistance of North European cultures to the spread of farming. PLoS ONE. 2015;10(4): e0121166. doi: 10.1371/journal.pone.0121166 25853888 PMC4390204

[29] Rigaud S, Manen C, García-Martínez de Lagrán I. Symbols in motion: flexible cultural boundaries and the fast spread of the Neolithic in the western Mediterranean. PLoS ONE. 2018;13(5): e0196488. doi: 10.1371/journal.pone.0196488 29715284 PMC5929525

[30] Vanhaeren M, d’Errico F. Aurignacian ethno-linguistic geography of Europe revealed by personal ornaments. J Archaeol Sci. 2006;33: 1105–1128. doi: 10.1016/j.jas.2005.11.017

[31] Bar-Yosef Mayer DE, Vandermeersch B, Bar-Yosef O. Shells and ochre in Middle Paleolithic Qafzeh Cave, Israel: indications for modern behavior. J Hum Evol. 2009;56: 307–314. doi: 10.1016/j.jhevol.2008.10.005 19285591

[32] Bar-Yosef Mayer DE, Groman-Yaroslavski I, Bar-Yosef O, Hershkovitz I, Kampen-Hasday A, Vandermeersch B, et al. On holes and strings: earliest displays of human adornment in the Middle Palaeolithic. PLoS ONE. 2020;15(7): e0234924. doi: 10.1371/journal.pone.0234924 32640002 PMC7343129

[33] Bouzouggar A, Barton N, Vanhaeren M, d’Errico F, Collcutt S, Higham T, et al. 82,000-year-old shell beads from North Africa and implications for the origins of modern human behavior. Proc Natl Acad Sci. USA 2007;104: 9964–9969. doi: 10.1073/pnas.0703877104 17548808 PMC1891266

[34] Henshilwood CS, d’Errico F, Vanhaeren M, van Niekerk K, Jacobs Z. Middle Stone Age shell beads from South Africa. Science. 2004;304: 403. doi: 10.1126/science.1095905 15087540

[35] Sehasseh EM, Fernandez P, Kuhn S, Stiner M, Mentzer S, Colarossi D, et al. Early Middle Stone Age personal ornaments from Bizmoune Cave, Essaouira, Morocco. Sci Adv. 2021;7(39): eabi8620. doi: 10.1126/sciadv.abi8620 34550742 PMC8457661

[36] Vanhaeren M, d’Errico F, Stringer C, James SL, Todd J, Mienis HK. Middle Palaeolithic shell beads in Israel and Algeria. Science. 2006;312: 1785–1788. doi: 10.1126/science.1128139 16794076

[37] Bar-Yosef Mayer DE. Shell beads of the Middle and Upper Palaeolithic: A review of the earliest Records. In: Mărgărit M, Boroneanț A, editors. Beauty and the eye of the beholder. Personal adornments across the millennia. Targoviște: Cetatea de Scaun; 2020. pp. 11–25.

[38] Kuhn SL, Stiner MC, Reese DS, Gülec E. Ornaments of the earliest Upper Paleolithic: new insights from the Levant. Proc Natl Acad Sci USA. 2001;98: 7641–7646. doi: 10.1073/pnas.121590798 11390976 PMC34721

[39] White R. Systems of personal ornamentation in the Early Upper Palaeolithic: methodological challenges and new observations. In: Mellars P, Boyle K, Bar-Yosef O, Stringer C, editors. Rethinking the human revolution. Cambridge: McDonald Institute for Archaeological Research; 2007. pp. 287–302.

[40] Richter T, Garrard AN, Allcock S, Maher LA. Interaction before agriculture: exchanging material and sharing knowledge in the Final Pleistocene Levant. Camb Archaeol J. 2011;21(1): 95–114. doi: 10.1017/S0959774311000060

[41] Wyllie C, Hole F. Personal adornment in the Epi-Paleolithic of the Levant. In: Matthews R, Curtis J, editors. Proceedings of the 7th International Congress on the Archaeology of the Ancient Near East, 2010, Vol. 3. Wiesbaden: Harrassowitz Verlag Publishers; 2012. pp. 707–717.

[42] Bar-Yosef Mayer DE. *Dentalium* shells used by hunter-gatherers and pastoralists in the Levant. Archaeofauna. 2008;17: 103–110.

[43] Davin L. La parure du Natoufien ancien en contexte funéraire. Reconstitution des chaînes opératoires à Mallaha (Eynan), Israël. Ph.D. dissertation, Université de Paris I. 2019. Available from: https://tel.archives-ouvertes.fr/tel-02932302

[44] Kurzawska A, Bar-Yosef Mayer DE, Mienis HK. Scaphopod Shells in the Natufian culture. In: Bar-Yosef O, Valla FR, editors. Natufian foragers in the Levant: terminal Pleistocene social changes in Western Asia. International Monographs in Prehistory, Archaeological Series 19. Ann Arbor, Michigan; 2013. pp. 611–621.

[45] Bar-Yosef O, Meadow RH. The origins of agriculture in the Near East. In: Price TD, Gebauer AB, editors. Last hunters, first farmers: new perspectives on the prehistoric transition to agriculture. Santa Fe: School of American Research Press; 1995. pp. 39–94.

[46] Goring-Morris AN, Belfer-Cohen A. Neolithization processes in the Levant: the outer envelope. Curr Anthropol. 2011;52(S4) The Origins of Agriculture: New Data, New Ideas: S195-S208. doi: 10.1086/658860

[47] Kuijt I, Goring-Morris AN. Foraging, farming, and social complexity in the Pre-Pottery Neolithic of the Southern Levant: a review and synthesis. J World Prehist. 2002;16(4): 361–440. doi: 10.1023/A:1022973114090

[48] Munro ND, Bar-Oz G, Meier J. Sapir-Hen L, Stiner MC, Yeshurun R. The Emergence of Animal Management in the Southern Levant. *Sci Rep* 8, 9279 (2018). doi: 10.1038/s41598-018-27647-z 29915348 PMC6006362

[49] Twiss KC. The Neolithic of the Southern Levant. Evol Anthropol. 2007;16: 24–35. doi: 10.1002/evan.20113

[50] Bar-Yosef DE. Changes in the selection of marine shells during the transition from the Natufian to the Neolithic. In: Bar-Yosef O, Valla FR, editors. The Natufian culture in the Levant. International Monographs in Prehistory, Archaeological Series 1. Ann Arbor, Michigan; 1991. pp. 629–636.

[51] Bar-Yosef Mayer DE. Neolithic shell bead production in Sinai. J Archaeol Sci. 1997;24: 97–111. doi: 10.1006/jasc.1995.0097

[52] Bar-Yosef Mayer DE. The exploitation of shells as beads in the Palaeolithic and Neolithic of the Levant. Paléorient. 2005;31(1): 176–185. doi: 10.3406/paleo.2005.4796

[53] Belfer-Cohen A, Goring-Morris AN. Becoming farmers: the inside story. Curr Anthropol. 2011;52(S4) The Origins of Agriculture: New Data, New Ideas: S209-S220. doi: 10.1086/658861

[54] Grosman L. The Natufian chronological scheme–new insights and their implications. In: Bar-Yosef O, Valla FR, editors. Natufian foragers in the Levant: terminal Pleistocene social changes in Western Asia. International Monographs in Prehistory, Archaeological Series 19. Ann Arbor, Michigan; 2013. pp. 622–637.

[55] Bar-Yosef O, Belfer-Cohen A. The Levantine ‘‘PPNB” interaction sphere. In: Hershkovitz I, editor. People and culture in change: Proceedings of the second symposium on Upper Paleolithic, Mesolithic and Neolithic populations of Europe and the Mediterranean Basin. BAR int. ser. 508(i). Oxford: Archaeopress; 1989. pp. 59–72.

[56] Bar-Yosef O, Bar-Yosef Mayer DE. Early Neolithic Tribes in the Levant. In: Parkinson WA, editor. The archaeology of tribal societies. International Monographs in Prehistory, Archaeology Series 15. Ann Arbor, Michigan; 2002. pp. 340–371.

[57] Belfer-Cohen A, Hovers E. Prehistoric perspectives on “others” and “strangers”. Front Psychol. 2020;10: 3063. doi: 10.3389/fpsyg.2019.03063 32038416 PMC6985552

[58] Bocquet-Appel JP. The agricultural demographic transition during and after the agriculture inventions. Curr Anthropol. 2011;52: S497–S510. doi: 10.1086/659243

[59] Coward F. Small worlds, material culture and ancient Near Eastern social networks. Proc Br Acad. 2010;158: 453–484. doi: 10.5871/bacad/9780197264522.003.0021

[60] Kuijt I. Life in Neolithic farming communities. Social organization, identity, and differentiation. New York: Kluwer Academic/Plenum Publishers; 2000.

[61] Asouti E. Beyond the Pre-Pottery Neolithic B interaction sphere. J World Prehist. 2006; 20: 87–126. doi: 10.1007/s10963-007-9008-1

[62] Goring-Morris AN, Belfer-Cohen A. Highlighting the PPNB in the Southern Levant. Neo-Lithics. 2020;20: 3–22.

[63] Barzilai O. Social complexity in the Southern Levantine PPNB as reflected through lithic studies–the bidirectional blade industries. BAR int. ser. 2180. Oxford: Archaeopress; 2010.

[64] Gopher A. Arrowheads of the Neolithic Levant: a seriation analysis. Winona lake: Eisenbrauns; 1994.

[65] Watkins T. Supra-regional networks in the Neolithic of Southwest Asia. J World Prehist. 2008;21: 139–171. doi: 10.1007/s10963-008-9013-z

[66] Goring-Morris AN. A PPNB settlement at Kfar Hahoresh in Lower Galilee: a preliminary report of the 1991 season. Mitekufat Haeven: Journal of the Israel Prehistoric Society. 1991;24: 77–101.

[67] Goring-Morris AN. The quick and the dead: the social context of Aceramic Neolithic mortuary practices as seen from Kfar HaHoresh. In: Kuijt I, editor. Life in Neolithic farming communities. Social organization, identity, and differentiation. New York: Kluwer Academic/Plenum Publishers; 2000. pp. 103–135.

[68] Goring-Morris AN. Life, death and the emergence of differential status in the Near Eastern Neolithic: evidence from Kfar HaHoresh, Lower Galilee, Israel. In: Clark J, editor. Archaeological perspectives on the transmission and transformation of culture in the Eastern Mediterranean. Oxford: CBRL & Oxbow Books; 2005. pp. 89–105.

[69] Goring-Morris AN, Goren Y, Horwitz LK, Hershkovitz I, Lieberman R, Sarel J, et al. The 1992 season of excavations at the Pre-Pottery Neolithic B settlement of Kefar HaHoresh. Mitekufat Haeven: Journal of the Israel Prehistoric Society. 1994–5;26: 74–121.

[70] Goring-Morris AN, Burns R, Davidzon A, Eshed V, Goren Y, Hershkovitz I, et al. The 1997 season of excavations at the mortuary site of Kfar HaHoresh, Galilee, Israel. Neo-Lithics. 1998;3/98: 1–4.

[71] Goring-Morris AN, Ashkenazi H, Barzilai O, Birkenfeld M, Eshed V, Goren Y, et al. The 2007–8 excavation seasons at Pre-Pottery Neolithic B Kfar HaHoresh, Israel. Antiquity Project Gallery. 2008;82(318).

[72] Goring-Morris AN, Horwitz LK. Funerals and feasts during the Pre-Pottery Neolithic B of the Near East. Antiquity. 2007;81(314): 902–919. doi: 10.1017/S0003598X00095995

[73] Goring-Morris AN, Boaretto E, Weiner S. Radiometric dating of the PPNB mortuary site of Kfar HaHoresh, Lower Galilee, Israel: problems and preliminary results. Mitekufat Haeven: Journal of the Israel Prehistoric Society. 2001;31: 213–217.

[74] Birkenfeld M. Changing systems: Pre-Pottery Neolithic B settlement patterns in the Lower Galilee, Israel. Studies in early Near Eastern production, subsistence, and environment 21. ex oriente: Berlin; 2018.

[75] Birkenfeld M, Goring-Morris AN. ’Out of sight’: the role of Kfar HaHoresh within the PPNB landscape of the Lower Galilee, Israel. Archaeological Review from Cambridge, Seen and Unseen Spaces. 2015;30(1): 7–16.

[76] Eshed V, Hershkovitz I, Goring-Morris AN. A re-evaluation of burial customs in the Pre-Pottery Neolithic B in light of paleodemographic analysis of the human remains from Kfar HaHoresh, Israel. Paléorient. 2008;34(1): 91–103. doi: 10.3406/paleo.2008.5234

[77] Goren Y, Goring-Morris AN, Segal I. The technology of skull modelling in the Pre-Pottery Neolithic B (PPNB): regional variability, the relation of technology and iconography and their archaeological implications. J Archaeol Sci. 2001;28: 671–690. doi: 10.1006/jasc.1999.0573

[78] Simmons TL, Goring-Morris AN, Horwitz LK. “What ceremony else?” Taphonomy and the ritual treatment of the dead in the Pre-Pottery Neolithic B mortuary complex at Kfar HaHoresh, Israel. In: Faerman M, Horwitz LK, Kahana T, Zilberman U, editors. Faces from the past: diachronic patterns in the biology and health status of human populations from the eastern Mediterranean. BAR int. ser. 1603. Oxford: Archaeopress; 2007. pp. 1–27.

[79] Horwitz KL, Goring-Morris AN. Animals and ritual during the Levantine PPNB: a case study from the site of Kfar Hahoresh, Israel. Anthropozoologica. 2004;39(1): 165–178.

[80] Meier JS, Goring-Morris AN, Munro N. Aurochs bone deposits at Kfar HaHoresh and the southern Levant across the agricultural transition. Antiquity. 2017;91(360): 1469–1483. doi: 10.15184/aqy.2017.179

[81] Goren Y, Goring-Morris AN. Early Pyrotechnology in the Near East: experimental lime-plaster production at the Pre-Pottery Neolithic B site of Kfar HaHoresh, Israel. Geoarchaeology. 2008;23(6): 779–798. doi: 10.1002/gea.20241

[82] Barzilai O, Goring-Morris AN. Blade caches in the southern Levant. in: Astruc L, Binder D, Briois F, editors. Technical systems and Near Eastern PPN communities. Antibes: Éditions APDCA; 2007. pp. 277–294.

[83] Barzilai O, Goring-Morris AN. Bidirectional blade production at the PPNB site of Kfar HaHoresh: The techno-typological analysis of a workshop dump. Paléorient. 2010;36(2): 5–34. doi: 10.3406/paleo.2010.5386

[84] Davidzon A, Goring-Morris AN. Knapping in the graveyard: a refitted naviform sequence from Kfar HaHoresh, Lower Galilee, Israel. In: Astruc L, Binder D, Briois F, editors. Technical systems and Near Eastern PPN Communities. Antibes: Éditions APDC; 2007. pp. 295–309.

[85] Goring-Morris AN. Aspects of the PPNB lithic assemblage from Kfar HaHoresh, Near Nazareth, Israel. In: Gebel HGK, Kozlowski SK, editors. Neolithic chipped lithic industries of the Fertile Crescent. Proceedings of the first workshop on PPN chipped lithic industries, SENEPSE 1. Berlin: ex oriente; 1994. pp. 427–444.

[86] Delerue S. L’Obsidienne dans le processus de Neolithisation du Proche-Orient (12,000–6,500 av. J.–C. cal). Ph.D. dissertation, Maison de l’Archeologie, Bordeaux 3. 2007.

[87] Bar-Yosef Mayer DE, Porat N. Green stone beads at the dawn of agriculture. Proc Natl Acad Sci USA. 2008;105(25): 8548–8551. doi: 10.1073/pnas.0709931105 18559861 PMC2438400

[88] Birkenfeld M, Goring-Morris AN. Stratigraphy and spatial analysis at Pre-Pottery Neolithic B Kfar HaHoresh, Israel: using GIS applications in inter-site analyses. In: García A, García J, Maximiano A, Ríos-Garaizar J, editors. Debating spatial archaeology. Santander: Instituto Internacional de Investigaciones Prehistóricas de Cantabria; 2014. pp. 65–79.

[89] Garrard AN. The Epipalaeolithic and Pre-Pottery Neolithic of Lebanon. In: Enzel Y and Bar-Yosef O, editors. Quaternary of the Levant: environments, climate change, and humans. Cambridge: Cambridge University press; 2017. pp. 691–697.

[90] Barzilai O. The bidirectional blade industries of the southern Levant. In: Borrell F, Ibáñez JJ, Molist M, editors. Stone tools in transition: from hunter-gatherers to farming societies in the Near East. Barcelona: Universitat Autònoma de Barcelona Press; 2013. pp. 59–72.

[91] Goring-Morris AN, Belfer-Cohen A. The southern Levant (Cisjordan) during the Neolithic period. In: Steiner M, Killebrew AE, editors. The Oxford Handbook of the archaeology of the Levant (ca 8000–332 BCE). Oxford: Oxford University Press; 2014. pp. 147–169.

[92] Peroschi ME, Mailland F, Mailland I, Anati E. Nahal Karkom, a Pre-Pottery Neolithic B site in the Southern Negev, Israel: archaeometric analysis. Mediterr Archaeol Ar. 2017; 18(3): 169–193.

[93] Gopher A, Goring-Morris AN. Abu Salem: A Pre-Pottery Neolithic B Camp in the Central Negev Highlands, Israel. Bull Am Schools Orient Res. 1998;312: 1–20. doi: 10.2307/1357671

[94] Gopher A, Goring-Morris AN, Rosen SA. ‘Ein Qadis I: A Pre-Pottery Neolithic B Occupation in Eastern Sinai. ‘Atiqot. 1995;27: 15–33.

[95] Mienis HK. Molluscs from the Excavation of the Pre-Pottery Neolithic B Site of ‘Ein Qadis I, Sinai. ‘Atiqot. 1995; 27: 35–36.

[96] Spatz AJ. Ornamental shell beads as markers of exchange in the Pre-Pottery Neolithic B of the Southern Levant. In: Bar-Yosef Mayer DE, Bonsall C, Choyke AM, editors. Not just for show, the archaeology of beads, beadwork and personal ornaments. Oxford: Oxbow Books; 2017. pp. 69–80.

[97] Benz M. Comments on radiocarbon dates of Epipalaeolithic and Early Neolithic sites of the Near East. Cited 11 July 2022. In: PPND—The platform for Neolithic radiocarbon dates [internet]. Available from: http://www.exoriente.org/associated_projects/ppnd.php.

[98] Perlès C. Tempi of change: when soloists don’t play together. Arrhythmia in ’continuous’ change. J Archaeol Method Theory. 2013;20(2): 281–299.

[99] Perlès C. Ornaments and other ambiguous artifacts from Franchthi. Volume 1, The Palaeolithic and Mesolithic. Bloomington: Indiana University Press; 2018.

[100] Blatterer H. Mollusca of the Dahab region. Denisia. 2019;43: 1–480.

[101] Milstein D, Mienis HK, Rittner O. [A field guide to the molluscs of inland waters of the Land of Israel]. Israel Nature and Parks Authority: Jerusalem; 2012. Hebrew.

[102] Poppe GT, Goto Y. European seashells. Wiesbaden: Verlag Christa Hemmen; 1991.

[103] Sharabati D. Red Sea Shells. London: KPI; 1984.

[104] WoRMS: World Register of Marine Species [Internet]. Cited 11 July 2022. Available from: http://www.marinespecies.org/. doi: 10.14284/170

[105] Bonnardin S. From traces to function of ornaments: some Neolithic examples. In: Longo L, Skakun N, editors. Prehistoric technology 40 years later. BAR int. ser. 1783. Oxford: Archaeopress; 2007. pp. 297–308.

[106] Cristiani E, Borić D. 8500-year-old Late Mesolithic garment embroidery from Vlasac (Serbia): technological, use-wear and residue analyses. J Archaeol Sci. 2012;39: 3450–3489. doi: 10.1016/j.jas.2012.05.016

[107] d’Errico F, Jardon-Giner P, Soler Major B. Critères à base expérimentale pour l’étude des perforations naturelles et intentionnelles sur coquillages. In: Anderson PC, Beyries S, Otte M, Plisson H. Traces et fonction: les gestes retrouvés. ERAUL 50. Liége: Université de Liège; 1993. pp. 243–254.

[108] Langley MC, O‘Connor S. 6500-Year-old *Nassarius* shell appliqués in Timor-Leste: technological and use wear analyses. J Archaeol Sci. 2015;62: 175–192. doi: 10.1016/j.jas.2015.06.012

[109] Mărgărit M. Testing the endurance of prehistoric adornments: raw materials from the aquatic environment. J Archaeol Sci. 2016;70: 66–81. doi: 10.1016/j.jas.2016.04.009

[110] Vanhaeren M, d’Errico F, van Niekerk KL, Henshilwood CS, Erasmus RM. Thinking strings: additional evidence for personal ornament use in the Middle Stone Age at Blombos Cave, South Africa. J Hum Evol. 2013;64: 500–517. doi: 10.1016/j.jhevol.2013.02.001 23498114

[111] Hammer Ø, Harper DAT, Ryan PD. PAST: paleontological statistics software package for education and data analysis. Palaeontol Electronica. 2001;4(1): 9pp.

[112] Liiv I. Seriation and matrix reordering methods: An historical overview. Stat Anal Data Min. 2010;3: 70–91. doi: 10.1002/sam.10071

[113] Buttigieg PL, Ramette A. A guide to statistical analysis in microbial ecology: a community-focused, living review of multivariate data analyses. FEMS Microbiol Ecol. 2014;90: 543–550. doi: 10.1111/1574-6941.12437 Guide available from: https://sites.google.com/site/mb3gustame/ 25314312

[114] Hammer Ø, Harper DAT. Paleontological data analysis. Oxford: Blackwell Publishing; 2006.

[115] Saitou N, Nei M. The neighbor-joining method: a new method for reconstructing phylogenetic trees. Mol Biol Evol. 1987;4: 406–425. doi: 10.1093/oxfordjournals.molbev.a040454 3447015

[116] Stiner MC. Finding a common bandwidth: causes of convergence and diversity in Paleolithic beads. Biol Theory. 2014;9: 51–64. doi: 10.1007/s13752-013-0157-4

[117] Benz M, Gresky J, Alarashi H. Similar but different–Displaying social roles of children in burials. In: Alarashi H, Dessì RM, editors. L’art du paraître: apparences de l’humain, de la Préhistoire à nos jours. 40° Rencontres internationales d’archéologie et d’histoire de Nice. Nizza: APDCA; 2020. pp. 93–107.

[118] Cabral JP, Martins JMS. Archaeological *Glycymeris glycymeris* shells perforated at the umbo: natural or man-made holes? J Archaeol Sci: Reports. 2016;10: 474–482. doi: 10.1016/j.jasrep.2016.11.008

[119] Kubicka AM, Rosin ZM, Tryjanowski P, Nelson E. A systematic review of animal predation creating pierced shells: implications for the archaeological record of the Old World. PeerJ. 2017;5:e2903. doi: 10.7717/peerj.2903 28123913 PMC5244880

[120] Zuschin M, Stachowitsch M, Stanton RJ Jr. Patterns and processes of shell fragmentation in modern and ancient marine environments. Earth Sci Rev. 2003;63: 33–82. doi: 10.1016/S0012-8252(03)00014-X

[121] Bosch MD, Mannino MA, Prendergast AL, Wesselingh FP, O’Connell TC, Hublin JJ. Year-round shellfish exploitation in the Levant and implications for Upper Palaeolithic hunter-gatherer subsistence. J Archaeol Sci: Reports. 2018;21: 1198–1214.

[122] d’Errico F, Henshilwood C, Vanhaeren M, van Niekerk K. *Nassarius kraussianus* shell beads from Blombos Cave: evidence for symbolic behaviour in the Middle Stone Age. J Hum Evol. 2005;48(1): 3–24. doi: 10.1016/j.jhevol.2004.09.002 15656934

[123] d’Errico F, Vanhaeren M, Barton N, Bouzouggar A, Mienis HK, Richter D, et al. Additional evidence on the use of personal ornaments in the Middle Paleolithic of North Africa. Proc Natl Acad Sci USA. 2009;106(38): 16051–16056. doi: 10.1073/pnas.0903532106 19717433 PMC2752514

[124] Schechter HC. The use of shells as adornments among PPNB communities in the Mediterranean zone of the Southern Levant. Ph.D. dissertation, The Hebrew University of Jerusalem. 2022.

[125] Bar-Yosef Mayer DE. Temporal changes in shell bead technologies based on Levantine examples. In: Szabó K, Dupont C, Dimitrijevic V, Gastélum Gómez LG, Serrand N, editors. Archaeomalacology: shells in the archaeological record. Proceedings of the 11th ICAZ international conference, Paris. BAR int. ser. 2666. Oxford: Archeopress; 2014. pp. 91–100.

[126] Francis P. Experiments with early techniques for making whole shells into beads. Curr Anthropol. 1982;23(6): 713–714. doi: 10.1086/202925

[127] Lemonnier P. Technology. In: Thieberger N, editor. The Oxford Handbook of Linguistic Fieldwork. Oxford: Oxford university Press; 2012. pp. 298–316.

[128] Choyke AM. Shifting meaning and value through imitation in the European Late Neolithic. In: Biehl PF, Rassamakin YY, editors. Import and imitation in archaeology. Langenweißbach: Beier & Beran; 2008. pp. 5–21.

[129] Meehan BF. Shell Bed to Shell Midden. Canberra: The Australian institute of Aboriginal Studies; 1982.

[130] Al Nahar M. ‘Ain Ghazal and Wadi Shueib: Neolithic personal ornaments. In: Finlayson B, Makarewicz C, editors. Settlement, survey and stone. Essays on Near Eastern prehistory in honour of Gary Rollefson. Berlin: ex oriente; 2014. Pp. 243–256.

[131] Bar-Yosef Mayer DE. Towards a typology of stone beads in the Neolithic Levant. J Field Archaeol. 2013;38.2: 129–142. doi: 10.1179/0093469013Z.00000000043

[132] Wright K, Garrard A. Social identities and the expansion of stone bead-making in Neolithic Western Asia: New evidence from Jordan. Antiquity. 2003;77(296): 267–284. doi: 10.1017/S0003598X00092267

[133] Goren Y, Goldberg P, Stahl PW, Brinker UH. Petrographic thin sections and the development of Neolithic plaster production in Northern Israel. J Field Archaeol. 1991;18(1): 131–140.

[134] Goren Y, Segal I, Bar-Yosef O. Plaster artefacts and the interpretation of the Nahal Hemar Cave. Mitekufat Haeven: Journal of the Israel Prehistoric Society. 1993;25: 120–131.

[135] Kingery WD. Plaster beads. In: Bar-Yosef O, Alon D, editors. Nahal Hemar Cave. ‘Atiqot. 1988;18: 45–46.

[136] Werker E. Botanical identification of worked wood remains. In: Bar-Yosef O, Alon D, editors. Nahal Hemar Cave. ‘Atiqot. 1988;18: 73–75.

[137] Hermansen BD. Raw materials of the small finds industries. In: Nissen HJ, Muheisen M, Gebel HGK, editors. Basta I. The human ecology. Berlin: ex oriente; 2004. pp. 117–128.

[138] Recchi A, Gopher A. Birds and humans in the Holocene: the case of Qumran Cave 24 (Dead Sea, Israel). Acta Zoologica Cracoviensia. 2002;45(special issue): 139–150.

[139] Alarashi H. Shell beads in the Pre-Pottery Neolithic B in Central Levant: Cypraeidae of Tell Aswad (Damascus, Syria). MUNIBE Suplemento—Gehigarria 2010;31: 88–98.

[140] d’Errico F, Vanhaeren M, Van Niekerk K, Henshilwood CS, Erasmus RM. Assessing the accidental versus deliberate colour modification of shell beads: A case study on perforated *Nassarius Kraussianus* from Blombos Cave Middle Stone Age levels. Archaeometry. 2013;57(1): 51–76. doi: 10.1111/arcm.12072

[141] Perlès C, Vanhaeren M. Black *Cyclope neritea* marine shell ornaments in the Upper Palaeolithic and Mesolithic of Franchthi Cave, Greece: arguments for intentional heat treatment. J Field Archaeol. 2010;35(3): 298–307.

[142] Schechter HC, Bar-Yosef Mayer DE. Shells from EPPNB Nesher-Ramla (NRQN). In: Ullman M, editor. The Early Pre-Pottery Neolithic B site at Nesher-Ramla Quarry (NRQN), Israel. Haifa: The Zinman Institute of Archaeology; 2020. pp. 149–168.

[143] de Swaan A. Widening circles of identification: emotional concerns in sociogenetic perspective. Theory Cult Soc. 1995;12(2): 25–39. doi: 10.1177/026327695012002002

[144] Frumkin A, Langford B, Porat R. The Judean Desert—The major hypogene cave region of the Southern Levant. In: Klimchouk AN, Palmer A, De Waele JS, Auler A, Audra P, editors. Hypogene karst regions and caves of the world. Cave and karst systems of the world. Cham: Springer; 2017. pp. 463–477. doi: 10.1007/978-3-319-53348-3_28

[145] Steinberger Y, Vishnevetsky S, Barness G, Lavee H. Effects of topoclimatic gradient on soil dehydrogenase activity in a Judean desert ecosystem. Arid Soil Res Rehab. 1998; 12(4): 387–393. doi: 10.1080/15324989809381525

[146] Crowfoot Payne J. The flint industries of Jericho. In: Kenyon KM, Holland TA, editors. Excavations at Jericho. Vol. 5. London: The British School of Archaeology in Jerusalem and the British Academy; 1983. pp. 622–758.

[147] Marx E. Are there pastoral nomads in the Middle East? In: Bar-Yosef O, Khazanov A, editors. Pastoralism in the Levant. Monographs on World Archaeology 10. Madison: Prehistory Press; 1992. pp. 255–260.

[148] Goring-Morris AN. The Harifian of the Southern Levant. In: Bar-Yosef O, Valla FR, editors. The Natufian culture in the Levant. International Monographs in Prehistory, Archaeological Series 1. Ann Arbor Michigan; 1991. pp. 173–216.

[149] Levy TE. The emergence of specialized pastoralism in the southern Levant. World Archaeol. 1983;15: 15–36. doi: 10.1080/00438243.1983.9979882

[150] Goring-Morris AN. From foraging to herding in the Negev and Sinai: the Early to Late Neolithic transition. Paléorient. 1993;19(1): 65–89. 10.3406/paleo.1993.4584

[151] Henry DO, White JJ, Beaver JE, Kadowaki S, Nowell A, Cordova C, et al. The Early Neolithic site of Ayn Abu Nukhayla, Southern Jordan. Bull Am Schools Orient Res. 2003;330: 1–30. doi: 10.2307/1357837

[152] Schechter HC, Zutovski K, Eirikh-Rose A, Ashkenazi H, Gopher A. Shells from Nahal Yarmut 38. Poster presented at the 9th International Conference on the PPN Chipped and Ground Stone Industries of the Near East, Tokyo, November 12–16, 2019. doi: 10.13140/RG.2.2.33733.47844

[153] Goring-Morris AN, Goren Y, Horwitz LK, Bar-Yosef Mayer DE, Hershkovitz I. Investigations at an Early Neolithic Settlement in the Lower Galilee: results of the 1991 season at Kefar HaHoresh. ‘Atiqot. 1995;28: 37–62.

[154] Nissenbaum A, Connan J. Application of organic geochemistry to the study of Dead Sea asphalt in archaeological sites in Israel and Egypt. In: Pike S, Gittin S, editors. The practical impact of science on Near Eastern and Aegean archaeology. Wiener Laboratory Publications 3. London: Archetype publications; 1999. pp. 91–98.

[155] Khalaily H, Valla FR 2013. Obsidian in Natufian Context: The case of Eynan (Ain Mallaha), Israel. In: Bar-Yosef O, Valla FR, editors. Natufian foragers in the Levant: terminal Pleistocene social changes in Western Asia. International Monographs in Prehistory, Archaeological Series 19. Ann Arbor, Michigan; 2013. pp. 193–202.

[156] Burian F, Friedman E. The obsidian finds of the PPNB Site Nahal Lavan 109. Mitekufat Haeven: Journal of the Israel Prehistoric Society. 1988;21: 5–59. Hebrew. (Pp. 95*-98* in English).

[157] Burian F, Friedman E, Mintz E. Nahal Lavan 109 –A Pre-Pottery Neolithic site in the Western Negev, Israel. Festschrift für Gunter Samulla. Materialien zur vor-und frühgeschichte von Hessen 1999;8: 95–120.

[158] Garfinkel Y. Obsidian distribution and cultural contacts in the Southern Levant during the 7th Millennium cal. BC. In: Healey E, Campbell S, Maeda O, editors. The state of the stone. Terminologies, continuities and contexts in Near Eastern lithics. SENEPSE 13. Berlin: ex oriente; 2011. pp. 409–420.

[159] Khalaily H, Bar-Yosef O, Barzilai O, Boaretto E, Bocuentin F, Le Dosseur G, et al. Excavations at Motza in the Judean Hills and the Early Pre-Pottery Neolithic B in the Southern Levant. Paléorient. 2007;33(2): 5–37.

[160] Renfrew C, Dixon JE, Cann JR. Obsidian and early cultural contact in the Near East. Proceedings of the Prehistoric Society. 1966;32: 30–72. doi: 10.1017/S0079497X0001433X

[161] Renfrew C, Dixon JE, Cann JR. Further analysis of Near Eastern obsidians. Proceedings of the Prehistoric Society. 1969;34: 319–331. doi: 10.1017/S0079497X0001392X

[162] Renfrew C. Alternative models for exchange and spatial distribution. In: Earle TK, Ericson JE, editors. Exchange systems in prehistory. New York: Academic press; 1977. pp. 71–90. doi: 10.1016/B978-0-12-227650-7.50010–9

[163] Ibáñez JJ, Ortega D, Campos D, Khalidi L, Méndez V. Testing complex networks of interaction at the onset of the Near Eastern Neolithic using modelling of obsidian exchange. Interface Focus. 2015;12: 20150210. doi: 10.1098/rsif.2015.0210 25948614 PMC4590507

[164] Ibáñez JJ, Ortega D, Campos D, Khalidi L, Méndez V, Teira L. Developing a complex network model of obsidian exchange in the Neolithic Near East: Linear regressions, ethnographic models and archaeological data. Paléorient. 2016;42(2) Connections and Disconnections between the Northern and Southern Levant in the Late Prehistory and Protohistory (12th–mid-2nd mill, BC): 9–32. doi: 10.3406/paleo.2016.5718

[165] Ortega D, Ibañez JJ, Khalidi L, Méndez V, Campos D, Teira L. Towards a Multi-Agent-Based modelling of obsidian exchange in the Neolithic Near East. J Archaeol Method Theory. 2014;21: 461–485. doi: 10.1007/s10816-013-9196-1

[166] Alt KW, Benz M, Vach W, Simmons TL, Goring-Morris AN. Insights into the social structure of the PPNB site of Kfar HaHoresh, Israel, based on dental remains. PLoS ONE. 2015;10(9): e0134528. doi: 10.1371/journal.pone.0134528 26376321 PMC4573520

[167] Fernández E, Pérez-Pérez A, Gamba C, Prats E, Cuesta P, Anfruns J, et al. Ancient DNA analysis of 8000 B.C. Near Eastern farmers supports an Early Neolithic pioneer maritime colonization of mainland Europe through Cyprus and the Aegean Islands. PLoS Genet. 2014;10(6): e1004401. doi: 10.1371/journal.pgen.1004401 24901650 PMC4046922

[168] Alarashi H, Ortiz A, Molist M. Sea shells on the riverside: cowrie ornaments from the PPNB site of Tell Halula (Euphrates, northern Syria). Quat Int. 2018;490: 98–112. doi: 10.1016/j.quaint.2018.05.004

[169] Schechter HC, Getzov N, Khalaily H, Milevski I, Goring-Morris AN, Bar-Yosef Mayer DE. Exceptional shell depositions at PPNB Yiftahel. J Archaeol Sci: Reports. 2021;37: 102944. doi: 10.1016/j.jasrep.2021.102944

[170] Ridout-Sharpe J. Changing lifestyles in the northern Levant: Late Epipalaeolithic and Early Neolithic shells from Tell Abu Hureyra. Quat Int. 2015;390: 102–116. doi: 10.1016/j.quaint.2015.11.041

[171] de Contenson H, Cauvin MC, Curtois L, Ducos P, Dupeyron M, van Zeist W. Les objects de parure, coquille, nacre et os. in: Ramad: site néolithique en Damascène (Syrie) aux VIIIe et VIIe millénaires avant l’ère chrétienne. Beirut: Institut Français d’Archéologie du Proche-Orient; 2000. pp. 171–178.

[172] Reese DS. On cassid lips and helmet shells. Bull Am School Orient Res. 1989;275: 33–39. doi: 10.2307/1356877

[173] Biggs HEJ. On the mollusca collected during the excavations at Jericho, 1952–1958, and their archaeological significance. Man. 1963;63: 125–128.

[174] Marshall DN. Jericho bone tools and objects. In: Kenyon KM, Holland TA, editors. Excavations at Jericho IV. The British School of Archaeology in Jerusalem. Oxford: University Press; 1982. pp. 570–622.

[175] Gopher A, Lemorini C, Boaretto E, Carmi I, Barkai R, Schechter HC. Qumran Cave 24, a Neolithic-Chalcolithic site by the Dead Sea: a short report and some information on lithics. In: Borrell F, Ibáñez JJ, Molist M, editors. Stone tools in transition: from hunter-gatherers to farming societies in the Near East. Barcelona: Universitat Autònoma de Barcelona Press; 2013. pp. 101–114.

[176] Bocquentin F, Kodas E, Ortiz A. Headless but still eloquent! Acephalous skeletons as witnesses of Pre-Pottery Neolithic North-South Levant connections and disconnections. Paléorient. 2016;42(2) Connections and disconnections between the Northern and Southern Levant in the late prehistory and protohistory (12th—mid-2nd mill. BC): 33–52. doi: 10.3406/paleo.2016.5719

[177] Bonogofsky M. Neolithic plastered skulls and railroading epistemologies. Bull Am School Orient Res. 2003;331: 1–10. doi: 10.2307/1357755

[178] Croucher K. Getting ahead: exploring meanings of skulls in the Neolithic Near East. In: Bonogofsky M, editor. Skull collection, modification and decoration. BAR int. ser. 1589. Oxford: archaeopress; 2006. pp. 29–44.

[179] Kuijt I. Negotiating equality through ritual: a consideration of Late Natufian and Pre Pottery Neolithic A period mortuary practices. J Anthropol Archaeol. 1996;15: 313–336. doi: 10.1006/jaar.1996.0012

[180] Nigro L. Beheaded ancestors. Of skulls and statues in Pre-Pottery Neolithic Jericho. Scienze dell’Antichità. 2017;23(3): 3–30.

[181] Garfinkel Y. The life cycle of Pre-Pottery Neolithic B plastered skulls from the Southern Levant. In: Finlayson B, Makarewicz C, editors. Settlements, survey, and stone. Essays on Near Eastern prehistory in honour of Gary Rollefson. Berlin: ex oriente; 2014. pp. 145–158.

[182] Kuijt I. The regeneration of life: Neolithic structures of symbolic remembering and forgetting. Curr Anthropol. 2008;49(2): 171–197. doi: 10.1086/526097

[183] Milevski I, Khalaily H, Getzov N, Hershkovitz I. The plastered skulls and other PPNB Finds from Yiftahel, Lower Galilee (Israel). Paléorient. 2008;34(2): 37–46. doi: 10.3406/PALEO.2008.5255

[184] Slon V, Sarig R, Hershkovitz I, Khalaily H, Milevski I. The plastered skulls from the Pre-Pottery Neolithic B Site of Yiftahel (Israel)–A Computed Tomography-Based analysis. PLoS ONE. 2014;9(2): e89242. doi: 10.1371/journal.pone.0089242 24586625 PMC3929714

[185] Fletcher A. 2020. Finding and losing the person within: A Neolithic plastered skull from Jericho. In: Sparks R.T., Finlayson B., Wagemakers B., and Briffa J.M. (eds.), *Digging Up Jericho*: *Past*, *Present and Future*. Archaeopress: Summertown. Pp. 93–102.

[186] Kenyon KM. Excavations at Jericho III. The architecture and stratigraphy of the tell. London: British School of Archaeology in Jerusalem; 1981.

[187] Strouhal E. Five plastered skulls from Pre-Pottery Neolithic B Jericho: anthropological study. Paléorient. 1973;1(2): 231–247.

[188] Garstang J. City and necropolis (Fifth Report). Liverpool Annal Archaeol Anthropol. 1935;22: 143–184.

[189] Mellaart J. Excavations at Catal Hüyük, 1965: fourth preliminary report. Anatolian Studies. 1966;16: 165–191. doi: 10.2307/3642483

[190] Reese DS. The Çatalhöyük shells. In: Hodder I, editor. Inhabiting Çatalhöyük: reports from the 1995–99 seasons. London: British Institute at Ankara; 2005. pp. 123–128.

