# Supplementary material for: Making ties and social identities: Drawing connections between PPNB communities as based on shell bead typology
Source: PLoS One. 2023 Nov 28;18(11):e0289091. doi: 10.1371/journal.pone.0289091 (PMC10684082; doi:10.1371/journal.pone.0289091)
Supplement: S1 Table — (DOCX) [file pone.0289091.s001.docx]

**Table 1.** **PPNB sites in the Levant used for the construction of the typological list and for comparison.**

| **Site Name** | **Abbreviation** | **Environmental zone** | **Landscape unit/region** | **Quantitative information where available – number of marine, freshwater, and Holed shells** | **Distance from Mediterranean/Red Sea**  **(approximate, in km)** | **Comments** | **References** |
| --- | --- | --- | --- | --- | --- | --- | --- |
| Abu Ma'adi III | AM3 | Desert | Desert - south | Marine – 196  Holed marine – 65 | 280/50 |  | Bar-Yosef Mayer 1999 |
| Ain Abū Nukhayla | AAN | Desert | Desert - centre | Marine – 1303  Holed marine – 602 | 225/40 |  | Spatz et al. 2014; Spatz 2008 |
| Ain Ghazal | AGZ | Mediterranean | Mediterranean - south | Marine – 167  Holed marine – 94  Freshwater – 66  Holed freshwater – 13 | 110/290 | PPNB and PPNC strata | Current study and mention of shells in:  Al Nahar 2014; Kafafi and Rollefson 1995; Kafafi et al. 1990; Rollefson 1983; Rollefson and Leonard 1982; Rollefson and Simmons 1984, 1985, 1986; Rollefson and Kafafi 1996; Rollefson et al. 1991 |
| Aswad | ASW | Mediterranean | Mediterranean - centre | Holed marine – 43  Holed freshwater – 15 | 115/440 |  | Alarashi 2010, 2014; Maréchal 1995 |
| Basta | BST | Desert | Desert - centre | Marine – 3192  Holed marine – 2759  Freshwater – 21  Holed freshwater – 7 | 175/95 | PPNB and PPNC strata | Current study and mention of shells in:  Gebel and Starck 1985; Gebel et al. 1988; Hermansen 1991; Hermansen 2004b; Nissen et al. 1987 |
| Beidha | BD | Desert | Desert - centre | Marine – 691  Holed marine – 383 | 160/100 | PPNB strata | Current study and mention of shells in:  Kirkbride 1960, 1966, 1967 |
| Beisamoun | BSMN | Mediterranean | Mediterranean - centre | Marine – 50  Holed marine – 27  Holed freshwater – 56*^1^ | 37/400 | 2007-2016 seasons only, PPNB and PPNC strata | Current study and  Bocquentin et al. 2014; 2020 |
| Gebel Rubsha | GR | Desert | Desert - south | Marine – 139  Holed marine – 49 | 280/55 |  | Bar-Yosef 1989, 1999 |
| Jericho | JRC | Desert | Desert - north | Marine – 39*^2^  Holed marine – 28 | 70/260 | PPNB strata | Biggs 1963; Holland 1983; Talbot 1983  Additional mentions in Marshall 1982 |
| Kfar HaHoresh | KHH | Mediterranean | Mediterranean - centre | Marine – 2174  Holed marine – 548  Freshwater – 595  Holed freshwater – 13 | 25/350 |  | Current study and mention of shells in: Birkenfeld 2018: 125-126; Goring-Morris 1991a; Goring-Morris et al. 1994-5, 1995 |
| Motza | MTZ | Mediterranean | Mediterranean - south | Marine – 21  Holed marine – 9  Freshwater – 15  Holed freshwater – 3 | 35/250 | PPNB strata | Mienis 2011 |
| Nahal Yarmut 38 | NY38 | Mediterranean | Mediterranean - south | Marine – 373  Holed marine – 87  Freshwater – 26 | 32/240 |  | Current study and  Schechter et al. 2019 |
| Nesher-Ramla Quarry | NRQN | Mediterranean | Mediterranean - south | Marine – 223  Holed marine – 87  Freshwater – 4  Holed freshwater – 1 | 21/260 |  | Schechter and Bar-Yosef Mayer 2020; Ullman et al. 2021 |
| Qumran Cave 24 | QC24 | Desert | Desert - north | Marine – 7  Holed marine – 7  Freshwater – 1 | 75/250 | PPNB strata | Gopher et al. 2013; Schechter 2012 |
| Ramad | RMD | Mediterranean | Mediterranean - centre | Marine – 66  Holed marine – 53  Freshwater – *^3^ | 70/440 |  | Contenson et al. 2000: 171-174 |
| Shkārat Msaied | SM | Desert | Desert - centre | Marine – 378  Holed marine – 278 | 155/110 | 1999-2005 seasons only | Abu-Laban 2014 |
| Tell Abu Hureyra | TAH | Mediterranean | Mediterranean - north | Marine – 53  Holed marine – 49  Freshwater – 549 *^4^  Holed freshwater – 48 | 220/770 | PPNB strata | Ridout-Sharpe 2015 |
| Tell Halula | THL | Mediterranean | Mediterranean - north | Holed marine – 479  Holed freshwater – 5 | 175/820 |  | Alarashi 2014; Alarashi et al. 2018 |
| Ujrat el Mehd | UM | Desert | Desert - south | Marine – 3386  Holed marine – 1486 | 280/55 |  | Bar-Yosef 1989, 1999 |
| Wadi Jibba I | WJ1 | Desert | Desert - south | Marine – 212  Holed marine – 88 | 300/70 |  | Bar-Yosef 1989, 1999 |
| Wadi Jibba II | WJ2 | Desert | Desert - south | Marine – 101  Holed marine – 30 | 300/70 |  | Bar-Yosef 1989, 1999 |
| Wadi Shu’eib | WSB | Mediterranean | Mediterranean - south | Marine – 6  Holed marine – 3 | 90/280 | PPNB and PPNC strata | Current study |
| Wadi Tbeik | WT | Desert | Desert - south | Marine – 1283  Holed marine – 648  Freshwater – 1 (Holed) | 250/60 |  | Bar-Yosef 1989, 1999 |
| Yiftahel | YFT | Mediterranean | Mediterranean - centre | Marine – 201  Holed marine – 22  Freshwater – 68 | 25/355 | 2007-2008 season only, from PPNB strata | Schechter et al. 2021 |

*^1^ Tens of thousands of freshwater shells were retrieved during the excavations at Beisamoun, located within the Hula Lake flood plain and damaged by modern agricultural fishponds. The numbers here are only of artificially holed freshwater shells.

*^2^ Hundreds of marine shells were retrieved from Jericho, many from PPN layers. Due to unsorted stratigraphic issues regarding those layers, in this analysis we considered only shells that were strictly assigned to PPNB contexts. The numbers here do not include 20 unidentified shells and four freshwater *Unio* shells embedded in the eyes of plastered skulls and a plaster statue from Jericho.

*^3^ Two remarkable shell concentrations were found in Ramad level II, that were not counted. The one contained abundant burnt shell fragments and was interpreted as possible consumption waste or manufacturing debris. The other was made up of multiple small conical shells which may have been freshwater shells.

*^4^ 431 of these freshwater shells are bivalves interpreted as nutritional consumption waste. Most holed freshwater shells (88%) are gastropods.
