# Supplementary material for: Making ties and social identities: Drawing connections between PPNB communities as based on shell bead typology
Source: PLoS One. 2023 Nov 28;18(11):e0289091. doi: 10.1371/journal.pone.0289091 (PMC10684082; doi:10.1371/journal.pone.0289091)
Supplement: S2 Table — (DOCX) [file pone.0289091.s002.docx]

**Table 2.** **Shell bead typological list and the occurrence of each bead type in the sampled sites.**

|  | **THL** | **TAH** | **RMD** | **ASW** | **BSMN** | **YFT** | **KHH** | **AGZ** | **WSB** | **NRQ** | **MTZ** | **NY38** | **JRC** | **QC24** | **SM** | **BD** | **BST** | **AAN** | **WT** | **AM3** | **GR** | **UM** | **WJ1** | **WJ2** |  |
| --- | --- | --- | --- | --- | --- | --- | --- | --- | --- | --- | --- | --- | --- | --- | --- | --- | --- | --- | --- | --- | --- | --- | --- | --- | --- |
| 1GNU | *Glycymeris*. Naturally worn hole on umbo. |  | * | * | * | * | * | * | * | * | * | * | * | * |  | * | * | * |  |  | * |  |  |  |  |
| 1GGU | *Glycymeris*. Ground hole on umbo. |  |  |  |  |  | * | * | * |  |  |  |  |  |  | * | * |  |  |  |  |  | * | * | * |
| 1GGHBU | *Glycymeris*. Gouged/hammered/broken hole on umbo. |  | * |  | * | * |  | * |  |  |  |  |  |  |  | * |  |  |  |  |  |  |  |  |  |
| 1GTSD | *Glycymeris*. Transverse slit sawn on dome. |  |  |  |  |  |  | * |  |  |  |  |  |  |  |  |  |  |  |  |  |  |  |  |  |
| 1GMP1 | *Glycymeris*. Multiple perforation - two drilled holes, one on each side of the umbo/dome. | * |  |  |  |  |  |  |  |  |  |  |  |  |  |  |  |  |  |  |  |  |  |  |  |
| 1GMP2 | *Glycymeris*. Multiple perforation - ground on umbo and transverse slit on dome. |  |  |  |  |  |  | * |  |  |  |  |  |  |  |  |  |  |  |  |  |  |  |  |  |
| 1CNU | Cardiidae. Naturally worn hole on umbo. |  |  | * |  | * | * | * | * |  | * |  | * | * |  | * | * |  |  |  |  |  |  |  |  |
| 1CGU | Cardiidae. Ground hole on umbo. |  |  |  | * |  | * | * | * |  |  |  | * |  |  | * | * | * |  |  |  |  |  |  |  |
| 1CGHBU | Cardiidae. Gouged/hammered/broken hole on umbo. |  |  |  |  |  |  | * |  |  |  |  |  |  |  | * |  |  |  |  |  |  |  |  |  |
| 1C-SGU | Cardiidae. – shaped slit sawn on umbo and then ground. |  |  |  |  |  |  | * |  |  |  |  |  |  |  |  |  |  |  |  |  |  |  |  |  |
| 1C+SU | Cardiidae. + shaped slit hole sawn on umbo. |  |  |  |  |  |  | * |  |  |  |  |  |  |  |  |  |  |  |  |  |  |  |  |  |
| 1CGD | Cardiidae. Ground hole on dome. |  |  |  |  |  |  | * |  |  |  |  |  |  |  |  |  |  |  |  |  |  |  |  |  |
| 1CGHBD | Cardiidae. Gouged/hammered/broken hole on dome. |  |  |  |  | * |  |  |  |  |  |  |  | * |  |  |  |  |  |  |  |  |  |  |  |
| 1MGU | *Mactra*. Ground hole on umbo. |  |  |  |  |  |  | * |  |  |  |  |  |  |  |  |  |  |  |  |  |  |  |  |  |
| 1DGU | *Donax*. Ground hole on umbo. |  |  |  |  |  |  |  |  |  |  |  |  | * |  |  |  |  |  |  |  |  |  |  |  |
| 1SGU | *Spondylus*. Ground on umbo. |  |  |  |  |  |  |  |  |  |  |  |  |  |  |  | * |  |  |  |  |  |  |  |  |
| 1RMP1 | *Ruditapes*. Multiple perforation - two drilled holes, one on each side of the umbo. |  |  |  |  |  |  |  |  |  |  |  |  |  |  |  |  | * |  |  |  |  |  |  |  |
| 2CDR | Cowrie. Dorsum removed. | * | * | * | * | * | * | * | * | * | * | * | * | * | * | * | * | * | * | * | * | * | * | * | * |
| 2CDRF | Cowrie. Dorsum removed. With flattened extremities. |  |  |  |  |  |  | * |  |  |  |  |  | * |  |  |  |  |  |  |  |  |  |  |  |
| 2CDRLD1 | Cowrie. Dorsum removed. Columellar lip, with drilled hole at centre of lip. |  |  |  | * |  |  |  |  |  |  |  |  |  |  |  |  |  |  |  |  |  |  |  |  |
| 2CDRLD2 | Cowrie. Dorsum removed. Columellar lip, with drilled hole at anterior end. |  |  |  | * |  |  |  |  |  |  |  |  |  |  |  |  |  |  |  |  |  |  |  |  |
| 2CDRLK1 | Cowrie. Dorsum removed. Columellar lip, with knob at anterior end. |  |  |  |  |  |  | * |  |  |  |  |  |  |  |  |  |  |  |  |  |  |  |  |  |
| 2CDRLK2 | Cowrie. Dorsum removed. Columellar lip, with knobs at both ends. | * | * | * |  |  |  |  |  |  |  |  |  |  |  |  |  |  |  |  |  |  |  |  |  |
| 2DRLPE | Cowrie. Dorsum removed. Outer lip, with pointed ends and body abraded round (like a cassid lip). |  |  |  |  |  |  |  |  |  |  |  |  |  |  |  |  |  |  | * |  |  | * |  | * |
| 2CDCS1 | Cowrie. Dorsum complete. One dorsum end perforated by Sawing. |  |  |  |  |  |  | * |  |  |  |  | * |  |  |  | * |  |  | * |  |  | * |  |  |
| 2CDCGH1 | Cowrie. Dorsum complete. One dorsum end perforated by gouging/hammering. |  |  |  |  | * |  |  |  |  |  |  |  |  |  |  |  |  |  |  |  |  |  |  |  |
| 2CDCS2 | Cowrie. Dorsum complete. Both dorsum ends perforated by sawing. |  |  |  |  |  |  |  |  |  |  |  |  |  |  |  | * |  |  |  |  |  |  |  |  |
| 2CDCGH2 | Cowrie. Dorsum complete. Both dorsum ends perforated by gouging/hammering. |  |  |  |  |  |  |  |  |  |  |  |  |  |  | * |  |  |  |  |  |  |  |  |  |
| 2DCLG | Cowrie. Dorsum complete. Ventral face of columellar lip perforated by grinding. |  |  |  |  |  |  | * |  |  |  |  |  | * | * |  |  |  |  |  |  |  |  |  |  |
| 2DCLD+4 | Cowrie. Dorsum complete. Ventral face of columellar lip perforated by drilling. Additional four pecked holes on four corners of dorsum. |  |  |  | * |  |  |  |  |  |  |  |  |  |  |  |  |  |  |  |  |  |  |  |  |
| 2VDRG | *Volvarina*. Dorsum removed by grinding. |  |  |  |  |  |  | * |  |  |  |  |  |  |  |  |  |  |  | * | * |  | * |  |  |
| 3TDP | *Tritia gibbosula*. Dorsum perforated. |  |  |  |  | * |  | * | * |  |  | * |  |  |  |  |  | * |  |  |  |  |  |  |  |
| 3TDR | *Tritia gibbosula*. Dorsum removed. |  |  | * | * | * | * | * | * |  | * |  | * | * | * | * | * | * | * | * | * | * | * | * | * |
| 3TCGHB | *Tritia gibbosula*. Callus perforated by gouging/hammering or natural breakage. |  |  |  | * |  |  | * | * |  |  |  | * |  |  |  |  | * |  | * | * |  | * |  |  |
| 3TCG | *Tritia gibbosula*. Callus perforated by grinding. |  |  |  |  |  |  | * |  |  |  |  | * |  |  |  |  |  |  |  |  |  |  |  |  |
| 3TCSG | *Tritia gibbosula*. Callus perforated by sawing and grinding. |  |  |  |  |  |  | * |  |  |  |  |  |  |  |  |  |  |  |  |  |  |  |  |  |
| 3TDPC | *Tritia gibbosula*. Dorsum perforated and callus perforated. |  |  |  |  |  |  | * |  |  |  |  |  |  |  |  |  |  |  |  |  |  |  |  |  |
| 3TDRC | *Tritia gibbosula*. Dorsum removed and callus perforated. |  |  |  |  | * |  | * | * |  |  |  | * |  |  |  |  |  | * |  |  |  |  |  |  |
| 3TAR | *Tritia gibbosula*. Aperture ring. |  |  |  |  |  |  | * |  |  |  |  |  |  |  |  |  |  |  |  |  |  |  |  |  |
| 4CASN | *Columbella*. Apex/spire perforated by natural wear/broken. |  |  |  |  | * | * | * | * |  |  |  |  |  |  |  |  | * |  |  |  |  |  |  |  |
| 4CASG | *Columbella*. Apex/spire perforated by grinding. |  |  |  | * |  |  | * |  |  |  |  |  |  |  |  |  |  |  |  |  |  |  |  |  |
| 4CFGHB | *Columbella*. Front of shell perforated by gouging/hammering or natural breakage. |  |  |  |  |  |  |  |  |  |  |  |  |  |  |  |  | * |  |  |  |  |  |  |  |
| 4CFG | *Columbella*. Front of shell perforated by grinding. |  |  |  |  |  |  | * |  |  | * |  |  |  |  |  |  | * |  |  |  |  |  |  |  |
| 4CBP | *Columbella*. Back of the shell perforated. |  |  |  |  |  |  | * |  |  |  |  |  |  |  |  | * |  | * |  |  |  |  |  |  |
| 4CASF | *Columbella*. Both apex/spire and front of shell perforated. |  |  |  |  |  |  | * |  |  |  |  |  |  |  |  |  |  |  |  |  |  |  |  |  |
| 4CASB | *Columbella*. Both apex/spire and back of shell perforated. |  |  |  |  |  |  | * |  |  |  |  |  |  |  |  |  |  |  |  |  |  |  |  |  |
| 4PSASB | *Pisania striata*. Both apex/spire and back of shell perforated. |  |  |  |  | * |  |  |  |  |  |  |  |  |  |  |  |  |  |  |  |  |  |  |  |
| 4PSASFB | *Pisania striata*. Apex/spire, front, and back of shell perforated. |  |  |  |  |  |  | * |  |  |  |  |  |  |  |  |  |  |  |  |  |  |  |  |  |
| 4MFP | *Melanopsis*. Front of shell perforated. |  |  |  |  | * |  | * | * |  |  |  |  |  |  |  |  |  |  |  |  |  |  |  |  |
| 4MBP | *Melanopsis*. Back of shell perforated. |  | * |  |  |  |  |  | * |  |  | * |  |  |  |  |  | * |  |  |  |  |  |  |  |
| 4ETBP | *Euplica turturina.* Back of shell perforated. |  |  |  |  |  |  |  |  |  |  |  |  |  |  |  |  |  |  |  |  |  | * |  |  |
| 4EMFGHB | *Engina mendicaria*. Front of shell perforated by gouging/hammering or natural breakage. |  |  |  |  |  |  |  |  |  |  |  |  |  |  |  | * | * |  |  |  |  |  |  |  |
| 4EMFG | *Engina mendicaria*. Front of shell perforated by grinding. |  |  |  |  |  |  |  |  |  |  |  |  |  |  |  | * | * |  |  |  |  |  |  |  |
| 4EMASF | *Engina mendicaria*. Both apex/spire and front of shell perforated. |  |  |  |  |  |  |  |  |  |  |  |  |  |  |  | * | * |  |  |  |  |  |  |  |
| 4EMBP | *Engina mendicaria*. Back of shell perforated. |  |  |  |  |  |  |  |  |  |  |  |  |  |  | * |  |  |  | * | * |  | * |  |  |
| 4EMFB | *Engina mendicaria*. Both front and back of shell perforated. |  |  |  |  |  |  | * |  |  |  |  |  |  |  |  |  |  |  |  |  |  |  |  |  |
| 4AFG | *Ancilla.* Front of shell perforated by Grinding. |  |  |  |  |  |  |  |  |  |  |  |  |  |  |  |  | * |  |  | * |  | * | * | * |
| 4AFD | *Ancilla.* Front of shell perforated by drilling. |  |  |  |  |  |  |  |  |  |  |  |  |  |  |  |  |  |  | * |  |  |  |  |  |
| 4ABP | *Ancilla.* Back of shell perforated. |  |  |  |  |  |  |  |  |  |  |  |  |  |  |  |  |  |  |  |  |  | * |  |  |
| 4AASF | *Ancilla.* Both apex/spire and front of shell perforated. |  |  |  |  |  |  |  |  |  |  |  |  |  |  |  |  |  |  | * |  |  |  |  |  |
| 4MiFG | *Mitra.* Front of shell perforated by grinding. |  |  |  |  |  |  |  |  |  |  |  |  |  |  |  |  | * |  |  |  |  |  |  |  |
| 5CASN | *Conus*. Apex/spire perforated by natural wear or unspecified technology. |  |  | * |  | * |  | * | * |  |  | * |  | * | * |  |  | * | * | * | * | * | * | * | * |
| 5CASG | *Conus*. Apex/spire perforated by grinding. |  |  |  | * |  | * | * |  |  | * |  |  |  |  |  | * |  | * |  |  |  |  |  |  |
| 5CASGF | *Conus*. Apex/spire perforated. Ground facets on the shell body. |  |  |  |  |  |  |  |  |  |  |  |  |  |  |  |  | * |  |  |  |  |  |  | * |
| 5CTB | *Conus*. 'Top Bead'. |  |  | * |  | * |  | * | * |  |  |  |  |  |  | * | * | * | * | * | * |  | * |  |  |
| 5CBB | *Conus*. 'Barrel bead' - Apex/spire perforated and base ground or naturally worn |  |  |  |  |  |  | * |  |  |  |  |  |  |  |  |  | * | * |  |  |  |  |  |  |
| 5CPB | *Conus*. Perforated on body whorl. |  |  | * |  |  |  |  |  |  |  |  |  |  |  |  |  | * |  |  |  |  |  |  |  |
| 5CSB | *Conus*. Slit sawn near base. |  |  |  |  |  |  |  |  |  |  |  |  |  |  | * |  |  |  | * |  | * |  |  |  |
| 5CASSB | *Conus*. Apex/spire perforated and slit sawn near base. |  |  |  |  |  |  |  |  |  |  |  |  |  |  |  |  |  |  | * |  |  |  |  |  |
| 6CFP | *Cerithium*. Front of shell perforated. |  |  |  |  |  |  | * |  |  |  |  |  |  |  |  |  |  |  |  |  |  |  |  |  |
| 6CBP | *Cerithium*. Back of shell perforated. |  |  |  |  |  |  |  |  |  |  |  |  |  |  |  | * | * |  |  |  |  |  |  |  |
| 6CSSP | *Cerithium*. Side of Spire perforated. |  |  |  |  |  |  |  |  |  | * |  |  |  |  |  |  |  |  |  |  |  |  |  |  |
| 6PCBP | *Pirenella conica.* Back of shell perforated. |  |  |  |  |  |  |  |  |  | * |  |  |  |  |  |  |  |  |  |  |  |  |  |  |
| 6TBG | *Terebra*. Lower body perforated by grinding. |  |  |  |  |  |  |  |  |  |  |  |  |  |  |  |  | * |  |  |  |  |  |  |  |
| 7HASPH | *Hexaplex*. Apex/spire perforated by hammering or breakage. |  |  |  |  |  |  | * |  |  |  |  |  |  |  |  |  |  |  |  |  |  |  |  |  |
| 7HBP | *Hexaplex*. (*Bolinus* at NY38). Back of shell perforated. |  |  |  | * | * |  | * |  |  | * |  | * |  |  |  |  |  |  |  |  |  |  |  |  |
| 7HASB | *Hexaplex*. Apex/spire and back of shell perforated. |  |  |  |  | * |  |  |  |  |  |  |  |  |  |  |  |  |  |  |  |  |  |  |  |
| 7HFBP | *Hexaplex*. Front and back of shell perforated. |  |  |  | * |  |  | * |  |  |  |  |  |  |  |  |  |  |  |  |  |  |  |  |  |
| 7HGIS+ | *Hexaplex*. Ground from apex to last whorl on back of shell creating an inclined perforated surface with an additional ground perforation on the front. |  |  |  |  |  |  | * |  |  |  |  |  |  |  |  |  |  |  |  |  |  |  |  |  |
| 7MAR | *Muricidae*. Aperture ring. |  |  |  |  |  |  | * |  |  |  |  |  |  |  |  |  |  |  |  |  |  |  |  |  |
| 7CSSG | *Chicoreus*. Side of spire perforated by grinding. |  |  |  |  |  |  |  |  |  |  |  |  |  |  |  |  | * |  |  |  |  |  |  |  |
| 7EASH | *Euthria*. Apex/spire perforated by hammering, breakage or natural wear. |  |  |  |  |  |  |  |  |  |  |  |  |  |  |  |  | * |  |  |  |  |  |  |  |
| 7SLSG | *Semicassis*. Labial side perforated by grinding. |  |  |  |  |  |  |  |  |  |  |  |  |  |  |  |  | * |  |  |  |  |  |  |  |
| 7SBP | Strombidae. Back of shell perforated. |  |  |  |  |  |  |  |  |  |  |  |  |  |  |  |  |  | * | * |  |  | * |  |  |
| 7SSB | Strombidae. Sawed slit on Back of shell near base. |  |  |  |  |  |  |  |  |  |  |  |  |  |  |  | * |  |  | * |  |  |  |  |  |
| 8NSW | *Nerita*. Side of whorl opposite aperture perforated. | * |  |  | * |  | * | * | * | * | * |  |  | * |  | * | * | * | * | * | * | * | * | * | * |
| 8NAR | *Nerita*. Aperture ring. |  |  |  |  |  |  |  |  |  |  |  |  |  |  |  |  |  |  | * |  |  | * | * |  |
| 8TSW | *Theodoxus*. Side of whorl opposite aperture perforated. |  |  |  | * | * |  | * | * |  |  |  |  |  |  |  |  | * |  |  |  |  |  |  |  |
| 8TBS | *Theodoxus/Neritina*. Back of shell behind aperture perforated. |  | * |  | * | * |  | * | * |  | * |  |  |  |  |  |  | * |  | * |  |  |  |  |  |
| 8TSWBS | *Theodoxus*. Side of whorl opposite aperture and back of shell behind aperture perforated. |  |  |  |  |  |  |  | * |  |  |  |  |  |  |  |  |  |  |  |  |  |  |  |  |
| 8CSW | *Tritia neritea*. Side of whorl opposite aperture perforated. |  |  | * |  |  |  |  |  |  |  |  |  |  |  |  |  | * |  |  |  |  | * |  |  |
| 8CBS | *Tritia neritea.* Back of shell behind aperture perforated. |  |  |  |  |  |  |  |  |  |  |  |  |  |  |  |  |  |  |  |  |  |  |  | * |
| 9NBWGH | *Neverita*. Body whorl perforated by gouging/hammering or natural breakage. |  |  |  |  |  |  |  |  |  | * |  |  | * |  |  |  |  |  |  |  |  |  |  |  |
| 9NBWS | *Neverita*. Body whorl perforated by sawn slit. |  |  |  |  |  |  | * |  |  |  |  |  |  |  |  |  |  |  |  |  |  |  |  |  |
| 9NASS | *Neverita*. Apex/spire perforated by sawn slit. |  |  |  |  |  |  |  |  |  |  |  |  | * |  |  |  |  |  |  |  |  |  |  |  |
| 9PBWGH | *Polinices*. Body whorl perforated by gouging/hammering or natural breakage. |  |  |  |  | * |  |  |  |  |  |  |  |  |  |  | * | * |  |  |  |  | * | * |  |
| 9PBWG | *Polinices*. Body whorl perforated by grinding. |  |  |  |  |  |  |  |  |  |  |  |  |  |  |  | * | * |  |  | * |  |  |  |  |
| 9MBWP | *Mammilla* (former *Polinices melanostoma and P. simiae*). Body whorl perforated. |  |  |  |  |  |  |  |  |  |  |  |  |  |  |  |  |  |  | * |  |  | * | * | * |
| 9NBWP | *Notocochlis* (former *Natica gualteriana*). Body whorl perforated. |  |  |  |  |  |  |  |  |  |  |  |  |  |  |  |  |  |  | * | * |  | * |  |  |
| 9OSSGH | *Phorcus turbinatus*. Side of Spire above aperture perforated by gouging/hammering or natural breakage. |  |  |  | * |  |  |  |  |  |  |  |  |  |  |  |  |  |  |  |  |  |  |  |  |
| 9CBWGH | *Clanculus*. Body whorl perforated by gouging/hammering or natural breakage. |  |  |  |  |  |  |  |  |  |  |  |  |  |  |  | * | * |  | * | * |  | * | * |  |
| 9HBWGH | *Heliacus*. Body whorl perforated by gouging/hammering or natural breakage. |  |  |  |  |  |  |  |  |  |  |  |  |  |  |  |  |  |  |  |  |  |  | * |  |
| 10CLDS | Cassid-lip. Drilled holes. Smooth body. |  |  |  |  | * |  | * | * |  |  |  |  |  |  |  |  | * |  |  |  |  |  |  |  |
| 10CLDC | Cassid-lip. Drilled holes. Carved body. |  |  |  |  |  |  | * |  |  |  |  |  | * |  |  |  |  |  |  |  |  |  |  |  |
| 10CLK | Cassid-lip. Knob ends. |  |  |  |  |  |  | * | * |  |  |  |  |  |  |  | * |  |  |  |  |  |  |  |  |
| 11DB1S | Disc bead. Single hole. Perforated by sawing. |  |  |  |  |  |  | * |  |  |  |  |  |  |  |  |  |  |  |  |  |  |  |  |  |
| 11DB1D | Disc bead. Single hole. Perforated by drilling. |  | * |  |  |  |  | * | * |  |  |  |  |  |  |  |  | * |  |  |  |  |  |  |  |
| 11DB2D | Disc bead. Two holes. Perforated by drilling. |  |  |  |  |  |  |  | * |  |  |  |  |  |  |  |  | * |  |  |  |  |  |  |  |
| 11DBMP | Disc bead. Mother-of-Pearl. |  |  |  |  |  |  |  | * |  |  |  |  | * |  |  |  | * |  |  |  |  |  |  |  |
| 12MPUN | MoP. Mention of additional artefacts without description or unknown outline. |  |  |  |  |  |  |  |  |  |  |  |  |  |  |  | * |  |  | * | * |  | * |  |  |
| 12MPV1 | MoP (Unionidae). Retaining partial shape of valve. Single perforation gouged/hammered to the right of the umbo. |  |  |  |  | * |  | * |  |  |  |  |  |  |  |  |  |  |  |  |  |  |  |  |  |
| 12MPV2 | MoP (Unionidae). Retaining partial shape of valve. Double perforation on umbo area. | * | * |  | * |  |  |  | * |  |  |  |  |  |  |  |  |  |  |  |  |  |  |  |  |
| 12MPRo1 | MoP. Round or oblong outline pendant. Single perforation. | * |  | * |  |  |  |  | * |  |  |  |  |  |  |  |  | * |  |  |  |  | * |  |  |
| 12MPRo2 | MoP. Round or oblong outline pendant. Double perforation. |  |  | * |  |  |  |  | * |  |  |  |  |  |  |  |  | * |  |  |  |  | * |  |  |
| 12MPRo3 | MoP. Round or oblong outline pendant. Multiple perforations. |  |  |  |  |  |  | * | * | * |  |  |  |  |  |  |  |  |  |  |  |  | * |  |  |
| 12MPCr | MoP. Crescent outline (or "eye") pendant. |  |  |  |  |  |  | * | * |  |  |  |  |  |  |  |  | * |  |  |  |  |  |  |  |
| 12MPTrp | MoP. Trapezoidal outline pendant. |  |  | * |  |  | * | * |  |  |  |  |  |  |  |  |  |  |  | * |  |  |  |  |  |
| 12MPRc | MoP. Rectangular outline pendant. One, two, or three holes. |  |  |  |  |  |  |  | * |  |  |  |  |  |  |  |  | * |  |  | * |  |  |  |  |
| 12MPTri | MoP. Triangular or rhomboid outline pendant. One or two holes. |  |  | * |  | * | * | * | * |  |  |  |  | * |  |  |  | * |  |  | * |  |  |  |  |
| 12MPEA | MoP. Elaborate or Amorphic shape pendant. One, two, or multiple holes. |  |  | * |  |  |  |  | * |  |  |  |  |  |  |  |  | * |  |  |  |  |  |  |  |
| 12MPRi | MoP. Ring. |  |  |  |  |  |  |  |  |  |  | * |  |  |  |  | * | * | * |  |  |  |  |  |  |
| 12MPRiP | MoP. Ring. With perforations. |  |  | * |  |  |  |  |  |  |  |  |  |  |  |  |  | * |  |  |  |  |  |  |  |
| 12MPB | MoP. Bangle. |  |  |  |  |  |  |  | * |  |  | * |  |  |  |  |  | * |  | * |  |  | * |  |  |
| 12MPBP | MoP. Bangle. With perforations. |  |  |  |  |  |  |  | * |  |  | * |  |  |  |  |  | * |  |  |  |  |  |  |  |
| 12MPPr | MoP. Rounded bead made on a Pearl. |  | * |  |  |  |  |  |  |  |  |  |  |  |  |  |  |  |  |  |  |  |  |  |  |
| 13SCAPH | Scaphopoda |  | * | * | * |  |  |  | * |  | * |  |  | * | * | * | * | * | * | * | * | * | * | * | * |
| 14TCB | *Tridacna*. Cylindrical beads. |  |  |  |  |  |  |  | * |  |  |  |  |  |  |  |  | * |  |  |  |  |  |  |  |
| 14TLB | *Tridacna.* Lentoid beads. |  |  |  |  |  |  |  |  |  |  |  |  |  |  |  |  | * |  |  |  |  |  |  |  |
| 14SPF | Conomurex. Perforated fragments |  |  |  |  |  |  |  |  |  |  |  |  |  |  |  |  | * |  |  |  |  |  |  |  |
| 14CSRi | *Conus*/conomurex. Ring |  |  |  |  |  |  |  |  |  |  |  |  |  |  |  |  | * |  |  |  |  |  |  |  |
| 14LCPF | *Lambis*/*Charonia*. Perforated fragments. |  |  |  |  |  |  |  |  |  |  |  |  |  |  |  |  | * |  |  | * |  |  |  |  |
| 14LB | *Lambis*. Bangle. |  |  |  |  |  |  |  |  |  |  |  |  |  |  |  |  |  |  | * |  |  |  |  |  |
| 14UNEP | Elongated pendants of an unidentified shell |  |  |  |  |  |  |  |  |  |  |  |  |  |  |  |  |  | * |  |  |  | * |  |  |
